# Autologous T cell therapy for MAGE-A4^+^ solid cancers in HLA-A*02^+^ patients: a phase 1 trial

**DOI:** 10.1038/s41591-022-02128-z

**Published:** 2023-01-09

**Authors:** David S. Hong, Brian A. Van Tine, Swethajit Biswas, Cheryl McAlpine, Melissa L. Johnson, Anthony J. Olszanski, Jeffrey M. Clarke, Dejka Araujo, George R. Blumenschein, Partow Kebriaei, Quan Lin, Alex J. Tipping, Joseph P. Sanderson, Ruoxi Wang, Trupti Trivedi, Thejo Annareddy, Jane Bai, Stavros Rafail, Amy Sun, Lilliam Fernandes, Jean-Marc Navenot, Frederic D. Bushman, John K. Everett, Derin Karadeniz, Robyn Broad, Martin Isabelle, Revashnee Naidoo, Natalie Bath, Gareth Betts, Zohar Wolchinsky, Dzmitry G. Batrakou, Erin Van Winkle, Erica Elefant, Armin Ghobadi, Amanda Cashen, Anne Grand’Maison, Philip McCarthy, Paula M. Fracasso, Elliot Norry, Dennis Williams, Mihaela Druta, David A. Liebner, Kunle Odunsi, Marcus O. Butler

**Affiliations:** 1 Department of Investigational Cancer Therapeutics, Division of Cancer Medicine, The University of Texas MD Anderson Cancer Center, Houston, TX USA; 2 Section of Medical Oncology, Division of Oncology, Siteman Cancer Center, Washington University School of Medicine, St. Louis, MO USA; 3 Adaptimmune, Abingdon, Oxfordshire UK; 4 Sarah Cannon Cancer Institute, Tennessee Oncology/One Oncology, Nashville, TN USA; 5 Department of Hematology/Oncology, Fox Chase Cancer Center, Philadelphia, PA USA; 6 Duke Cancer Institute, Duke University, Durham, NC USA; 7 Department of Sarcoma Medical Oncology, Division of Cancer Medicine, The University of Texas MD Anderson Cancer Center, Houston, TX USA; 8 Department of Thoracic-Head and Neck Medical Oncology, Division of Cancer Medicine, The University of Texas MD Anderson Cancer Center, Houston, TX USA; 9 Department of Stem Cell Transplantation and Cellular Therapy, Division of Cancer Medicine, The University of Texas MD Anderson Cancer Center, Houston, TX USA; 10 Adaptimmue, Philadelphia, PA USA; 11 Department of Microbiology, University of Pennsylvania, Philadelphia, PA USA; 12 Department of Medicine, Roswell Park Comprehensive Cancer Center, Buffalo, NY USA; 13 Sarcoma Medical Oncology, Moffitt Cancer Center, Tampa, FL USA; 14 Department of Internal Medicine, Division of Medical Oncology, and Department of Biomedical Informatics, Division of Computational Biology and Bioinformatics, Ohio State University, Columbus, OH USA; 15 University of Chicago Medicine Comprehensive Cancer Center, Chicago, IL USA; 16 Department of Medical Oncology and Hematology, Princess Margaret Cancer Centre, University of Toronto, Toronto, Canada

**Keywords:** Cancer immunotherapy, Head and neck cancer, Sarcoma, Ovarian cancer, T cells

## Abstract

Affinity-optimized T cell receptors can enhance the potency of adoptive T cell therapy. Afamitresgene autoleucel (afami-cel) is a human leukocyte antigen-restricted autologous T cell therapy targeting melanoma-associated antigen A4 (MAGE-A4), a cancer/testis antigen expressed at varying levels in multiple solid tumors. We conducted a multicenter, dose-escalation, phase 1 trial in patients with relapsed/refractory metastatic solid tumors expressing MAGE-A4, including synovial sarcoma (SS), ovarian cancer and head and neck cancer (NCT03132922). The primary endpoint was safety, and the secondary efficacy endpoints included overall response rate (ORR) and duration of response. All patients (*N* = 38, nine tumor types) experienced Grade ≥3 hematologic toxicities; 55% of patients (90% Grade ≤2) experienced cytokine release syndrome. ORR (all partial response) was 24% (9/38), 7/16 (44%) for SS and 2/22 (9%) for all other cancers. Median duration of response was 25.6 weeks (95% confidence interval (CI): 12.286, not reached) and 28.1 weeks (95% CI: 12.286, not reached) overall and for SS, respectively. Exploratory analyses showed that afami-cel infiltrates tumors, has an interferon-γ-driven mechanism of action and triggers adaptive immune responses. In addition, afami-cel has an acceptable benefit–risk profile, with early and durable responses, especially in patients with metastatic SS. Although the small trial size limits conclusions that can be drawn, the results warrant further testing in larger studies.

## Abstract (web summary)

In a phase 1 dose-escalation trial in patients with nine different types of solid tumors, MAGE-A4-specific T cells had an acceptable safety profile and exhibited an encouraging overall response rate in patients with synovial sarcoma.

## Main

Melanoma-associated antigen A4 (MAGE-A4) is a member of the MAGE protein family of cancer/testis antigens, with expression in healthy tissue restricted to immune-privileged sites^1^. MAGE-A4 is expressed in solid cancers, including synovial sarcoma (SS), myxoid/round cell liposarcoma (MRCLS), non-small-cell lung cancer (NSCLC), head and neck squamous cell carcinoma (HNSCC) and ovarian, urothelial, melanoma and gastroesophageal cancers^1–5^. MAGE-A4 is intracellularly processed, resulting in peptide fragments that are co-presented with human leukocyte antigens (HLAs) on the cell surface, forming epitopes that are weakly recognized by low-affinity natural T cell receptors (TCRs). Although immune checkpoint inhibitors have exhibited good clinical activity in patients with some MAGE-A4^+^ solid tumors, such as melanoma, other tumors, such as SS, may not respond as well^6,7^.

Afamitresgene autoleucel (afami-cel) is an autologous, specific peptide enhanced affinity receptor, T cell therapy transduced via a lentiviral vector to express a high-affinity and specific TCR targeted against a MAGE-A4_230−239_ peptide, GVYDGREHTV, presented by HLA-A*02 (ref. 6). This TCR has been shown to respond potently toward MAGE-A4 peptides presented on multiple common HLA-A2 alleles. Preclinical assessment demonstrated that afami-cel induces potent cytotoxic effects and effector cytokine release against multiple HLA-A*02/MAGE-A4 cancer cells^8^, supporting the first-in-human phase 1 trial of afami-cel (NCT03132922).

## Results

### Patients

HLA-A*02-eligible and MAGE-A4-eligible patients were enrolled from a multicenter, screening protocol study (NCT02636855). A total of 854 HLA-A*02-eligible patients proceeded to tumor MAGE-A4 testing: 225 were MAGE-A4^+^. This phase 1 trial used a 3 + 3 design, involving afami-cel dose escalation across dose Groups 1–3 and an expansion group. Dose ranges (total transduced cell number) were 0.08 × 10^9^ to 0.12 × 10^9^ cells (Group 1), 0.5 × 10^9^ to 1.2 × 10^9^ cells (Group 2), 1.2 × 10^9^ to 6.0 × 10^9^ cells (Group 3) and 1.2 × 10^9^ to 10 × 10^9^ cells (expansion group). Group 1 received cyclophosphamide (600 mg/m^2^/day) lymphodepletion (LD) chemotherapy on days −7, −6 and −5 and fludarabine (30 mg/m^2^/day) on days −7, −6 and −5. Group 2 received cyclophosphamide (600 mg/m^2^/day) LD chemotherapy on days −7, −6 and −5 and fludarabine (30 mg/m^2^/day) on days −7, −6 and −5. Group 3 received cyclophosphamide (600 mg/m^2^/day) LD chemotherapy on days −7, −6 and −5 and fludarabine (30 mg/m^2^/day) on days −7, −6, −5 and −4. Most patients in the expansion group (*n* = 22) received cyclophosphamide (600 mg/m^2^/day) LD chemotherapy on days −7, −6, and −5 and fludarabine (30 mg/m^2^/day) on days −7, −6, −5 and −4. Seven patients in the expansion group received the higher cyclophosphamide (1,800 mg/m^2^/day) LD chemotherapy on days −3 and −2 and combined with fludarabine (30 mg/m^2^/day) on days −5, −4, −3 and −2. Dose-limiting toxicities (DLTs) were evaluated before each dose escalation, with doses progressively increased to 1.2 × 10^9^ to 10.0 × 10^9^ cells in the expansion group. Eligible patients could receive a second cell infusion after disease progression after confirmed response.

Eligibility criteria included age ≥18 years to ≤75 years; histologically confirmed cancer diagnosis; positivity for at least one HLA-A*02 inclusion allele; MAGE-A4 RNA or protein expression in one or more tumor samples; and measurable disease according to Response Evaluation Criteria in Solid Tumors (RECIST) version 1.1 before LD chemotherapy (see Methods for full inclusion criteria). The sample size was not pre-specified and was based on clinical judgment. Up to 30 patients (including patients accrued during the dose-escalation phase) were treated in the dose-expansion phase, and up to an additional ten patients were treated in a radiation sub-study. The study was not statistically powered to evaluate safety or efficacy, and data are descriptive, with no formal hypothesis testing planned. The primary endpoints were adverse events (AEs), including serious AEs; laboratory assessments, including chemistry, hematology and coagulation; incidence of DLTs; determination of optimally tolerated dose range; and persistence of MAGE-A4^c1032T^ and replication-competent lentivirus over time. Secondary endpoints were overall response rate (ORR) confirmed by RECIST version 1.1; best overall response (BOR); time to response (TTR); duration of response (DoR); duration of stable disease (SD); progression-free survival (PFS); overall survival (OS); and presence of any of the following long-term follow-up (LTFU) AEs: new malignancies; new incidence or exacerbation of a pre-existing neurologic disorder and/or prior rheumatologic or other autoimmune disorder; new incidence of a hematologic disorder; opportunistic and/or serious infections; unanticipated illness and/or hospitalization deemed related to gene-modified cell therapy; and/or persistence of MAGE-A4^c1032T^ and replication-competent lentivirus over time. The exploratory endpoints included correlation of persistence, phenotype and functionality of transduced (afami-cel) and non-transduced T cells in the peripheral blood and/or tumor in response to treatment and safety; determination of target antigen expression, genes related to antigen processing/presentation and cell surface co-stimulatory ligands; and evaluation of serum cytokines (IL-6). The following exploratory endpoints were not analyzed: evaluation of anti-tumor antibodies or candidate biomarkers from plasma-derived exosomes and cell-free DNA. Owing to the preliminary nature of the data collected before the data cutoff, limited correlative analyses were reported relating to treatment response and safety. Two patients received a second infusion; they were non-responders. Therefore, ORR was not evaluated for the second infusion.

**Fig. 1:**
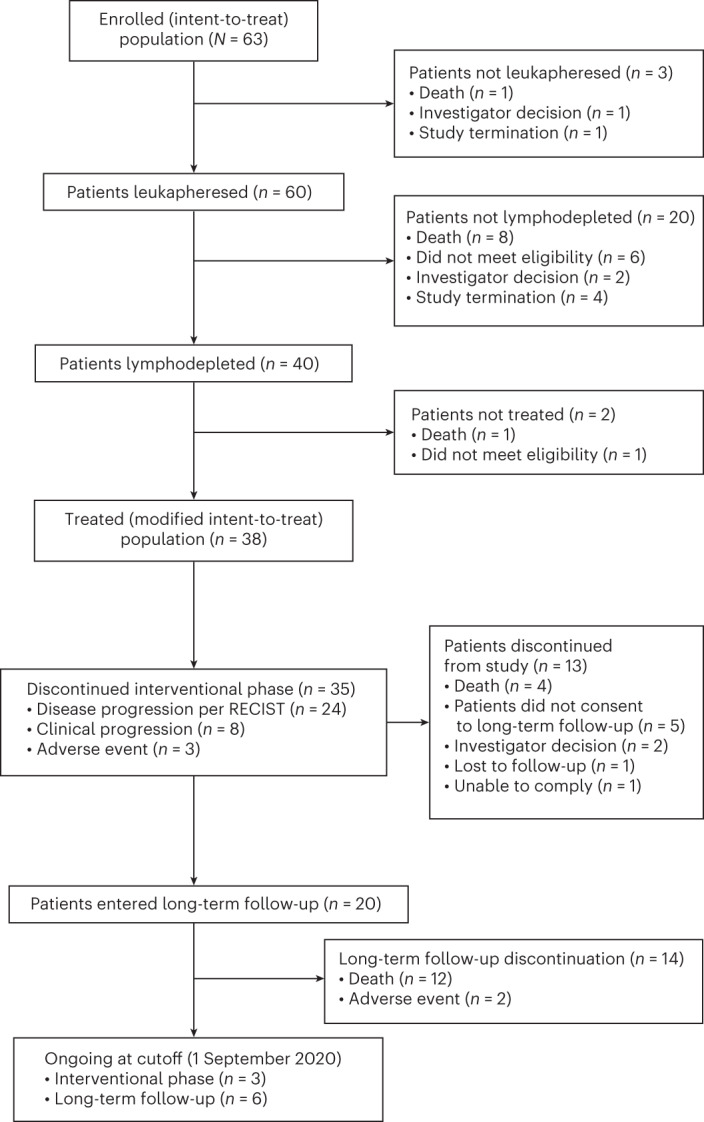
Study design and patient disposition. RECIST.

**Extended Data Fig. 1:**
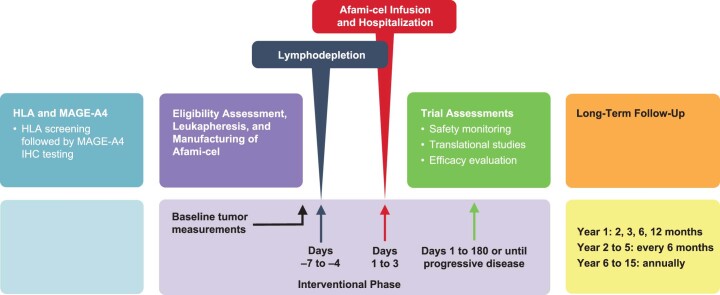
Study design. HLA, human leukocyte antigen; IHC, immunohistochemistry; MAGE-A4, melanoma-associated antigen A4.

**Extended Data Fig. 2:**
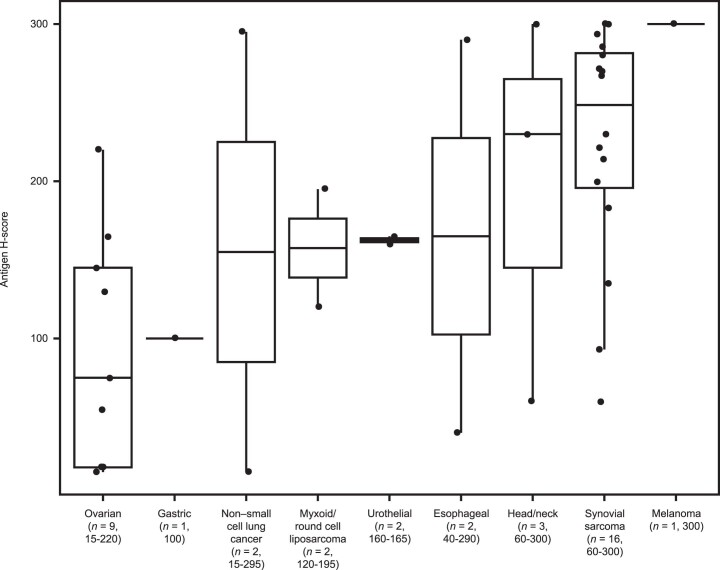
MAGE-A4 antigen H-score in the modified intent-to-treat population by tumor type. Individual MAGE-A4 antigen H-scores per tumor type. The overall median MAGE-A4 H-score across all nine tumor types was 189 (range: 15–300). Median MAGE-A4 H-score was highest at 249 in synovial sarcoma (range: 60–300); melanoma sub-group excluded due to a single evaluable patient. Box plots depict median as horizontal lines within boxes, with box bounds as the first and third quartiles. Dots represent individual data points. Lower whisker is the minimum value of the data within 1.5 times the interquartile range below the 25^th^ percentile. Upper whisker is the maximum value of the data within 1.5 times the interquartile range above the 75^th^ percentile. MAGE-A4, melanoma-associated antigen A4.

Sixty-three patients were deemed eligible and were enrolled into the intent-to-treat (ITT) population; 60 patients underwent leukapheresis; and 38 patients were treated with afami-cel (modified ITT (mITT) population) (Fig. 1 and Extended Data Fig. 1). Baseline patient characteristics are presented in Table 1 and Supplementary Tables 1 and 2. The first patient was enrolled on 5 July 2017; the last patient visit was 30 December 2019. Three patients did not undergo leukapheresis due to death, study termination and investigator decision (one each). Twenty patients underwent leukapheresis but did not receive LD chemotherapy or afami-cel due to death from disease under study (seven), death from unknown cause (one), not meeting eligibility criteria (six) or investigator decision (six). Two of the 40 patients who underwent LD chemotherapy did not receive afami-cel due to disease-related death or because they became ineligible after LD chemotherapy. The HLA-A2 and MAGE-A4 antigen scores in the mITT population are shown in Supplementary Table 1 and Extended Data Fig. 2. In the mITT population, three patients were treated in each dose-escalation group, and 29 patients were treated in the expansion group. The number (%) of patients with each indication are two (5.3) esophageal, one (2.6) gastric, three (7.9) head and neck, one (2.6) melanoma, two (5.3) NSCLC, nine (23.7) ovarian, two (5.3) urothelial, two (5.3) MRCLS and 16 (42.1) SS.

**Table 1:** Summary of patient characteristics (mITT population, *N* = 38)

Parameter	Category	Statistic	Overall (*N* = 38)
Age (years)		*N*	38
		Mean (standard deviation)	56.4 (12.59)
		Median	58.0
		Min, max	31, 78
Age categorization	<65 years	*n* (%)	28 (73.7)
	≥65 years	*n* (%)	10 (26.3)
Sex	Male	*n* (%)	22 (57.9)
	Female	*n* (%)	16 (42.1)
Ethnicity	Hispanic/Latino	*n* (%)	2 (5.3)
	Not Hispanic/Latino	*n* (%)	36 (94.7)
Race	White	*n* (%)	35 (92.1)
	Asian	*n* (%)	3 (7.9)
Primary tumor type	Esophageal	*n* (%)	2 (5.3)
	Gastric	*n* (%)	1 (2.6)
	Head and neck	*n* (%)	3 (7.9)
	Melanoma	*n* (%)	1 (2.6)
	NSCLC	*n* (%)	2 (5.3)
	Ovarian	*n* (%)	9 (23.7)
	Urothelial	*n* (%)	2 (5.3)
	MRCLS	*n* (%)	2 (5.3)
	SS	*n* (%)	16 (42.1)
ECOG score	0	*n* (%)	13 (34.2)
	1	*n* (%)	25 (65.8)
Time from initial diagnosis to T cell infusion (months)^a^		Mean (standard deviation)	51.4 (40.33)
		Median	40.4
		Min, max	7.3, 205.5
Prior lines of systemic therapy		Mean (standard deviation)	3.2 (1.88)
		Median	3.0
		Min, max	1, 8
Total transduced cells (×10^9^)		Mean (standard deviation)	6.1213 (3.422688)
		Median	6.4022
		Min, max	0.1, 9.9756

^a^Time from initial diagnosis to T cell infusion in months was calculated as: (T cell infusion date – date since initial diagnosis + 1) × (12/365.25).

max, maximum; min, minimum; *N*, total number of patients; *n*, number of patients.

At baseline, 58% of the mITT patients were male, and their median age was 58 years (range, 31–78). The median number of prior lines of systemic therapy was three (range, 1–8), including neoadjuvant and adjuvant therapies (Table 1 and Supplementary Table 2). Among patients with SS (Supplementary Table 3), the median age was 49 years (range, 31–76), and the median number of prior lines of treatment was three (range, 1–6), including ifosfamide (100%), anthracyclines (81%) and pazopanib (31%). MAGE-A4 expression was heterogenous within individual tumor types and across the nine solid tumor types evaluated. The median MAGE-A4 histoscore (H-score) was 189 (range, 15–300) across all patients and 249 (range, 60–300) in patients with SS.

Twenty-one patients received cytotoxic bridging chemotherapy (Supplementary Table 2). Twenty patients (53%) entered LTFU: 12 (32%) died of disease progression; two (5%) discontinued owing to AEs; and six (16%) remain in LTFU. Of the 18 patients who did not enter LTFU, three were being followed at the data cutoff; 13 left during the interventional phase; and two completed the interventional phase but did not enter LTFU.

### Safety

Toxicities determined by the Safety Review Committee as DLTs occurred in six patients and included cytopenias (four), aplastic anemia (one) and cerebrovascular accident (one); all DLTs occurred in the expansion group (median dose, 7.85 × 10^9^ transduced cells) after dose escalation completion. All 38 patients in the mITT group had Grade ≥3 treatment-emergent AEs (TEAEs) (Table 2); hematologic toxicities were the most common, including Grade ≥3 decreased counts for lymphocytes (97%), neutrophils (87%) and platelets (42%) and anemia (63%). Grade ≥3 febrile neutropenia and pancytopenia occurred in 32% and 11% of patients, respectively. Prolonged cytopenia, defined as Grade ≥3 neutropenia, anemia or thrombocytopenia persisting at week 4 after afami-cel treatment, occurred in 17 patients (45%), including nine (24%) with prolonged neutropenia. Serious treatment-related systemic infections were infrequent (3%).

**Table 2:** Incidence of TEAEs

Preferred term, *n* (%)	Group 1 (*n* = 3)		Group 2 (*n* = 3)		Group 3 + expansion group (*n* = 32)		Overall (*N* = 38)	
	All grades	Grade ≥ 3	All grades	Grade ≥ 3	All grades	Grade ≥ 3	All grades	Grade ≥ 3
Patients with any TEAEs	3 (100)	3 (100)	3 (100)	3 (100)	32 (100)	32 (100)	38 (100)	38 (100)
Lymphocyte count decreased	3 (100)	3 (100)	3 (100)	3 (100)	31 (96.9)	31 (96.9)	37 (97.4)	37 (97.4)
White blood cell count decreased	3 (100)	3 (100)	3 (100)	3 (100)	28 (87.5)	28 (87.5)	34 (89.5)	34 (89.5)
Neutrophil count decreased	3 (100)	3 (100)	3 (100)	3 (100)	27 (84.4)	27 (84.4)	33 (86.8)	33 (86.8)
Anemia	2 (66.7)	2 (66.7)	2 (66.7)	2 (66.7)	23 (71.9)	20 (62.5)	27 (71.1)	24 (63.2)
Fatigue	3 (100)	0	2 (66.7)	0	19 (59.4)	1 (3.1)	24 (63.2)	1 (2.6)
Nausea	3 (100)	0	2 (66.7)	0	19 (59.4)	0	24 (63.2)	0
Pyrexia	1 (33.3)	0	3 (100)	0	18 (56.3)	0	22 (57.9)	0
CRS	1 (33.3)	0	0	0	20 (62.5)	2 (6.3)	21 (55.3)	2 (5.3)
Platelet count decreased	2 (66.7)	1 (33.3)	1 (33.3)	1 (33.3)	18 (56.3)	14 (43.8)	21 (55.3)	16 (42.1)
Vomiting	2 (66.7)	0	2 (66.7)	0	15 (46.9)	1 (3.1)	19 (50.0)	1 (2.6)
Decreased appetite	3 (100)	0	2 (66.7)	0	11 (34.4)	2 (6.3)	16 (42.1)	2 (5.3)
Dyspnea	2 (66.7)	0	2 (66.7)	0	12 (37.5)	1 (3.1)	16 (42.1)	1 (2.6)
Hypophosphatemia	0	0	2 (66.7)	2 (66.7)	13 (40.6)	11 (34.4)	15 (39.5)	13 (34.2)
Diarrhea	2 (66.7)	0	0	0	12 (37.5)	0	14 (36.8)	0
Hypotension	0	0	1 (33.3)	1 (33.3)	13 (40.6)	3 (9.4)	14 (3.8)	4 (10.5)
Febrile neutropenia	1 (33.3)	1 (33.3)	2 (66.7)	2 (66.7)	9 (28.1)	9 (28.1)	12 (31.6)	12 (31.6)
Hyponatremia	2 (66.7)	2 (66.7)	1 (33.3)	0	9 (28.1)	6 (18.8)	12 (31.6)	8 (21.1)
Abdominal pain	2 (66.7)	0	2 (66.7)	0	7 (21.9)	1 (3.1)	11 (28.9)	1 (2.6)
Headache	0	0	3 (100)	0	7 (21.9)	0	10 (26.3)	0
Arthralgia	0	0	1 (33.3)	0	8 (25.0)	1 (3.1)	9 (23.7)	1 (2.6)
Aspartate aminotransferase increased	0	0	1 (33.3)	0	8 (25.0)	1 (3.1)	9 (23.7)	1 (2.6)
Chills	0	0	1 (33.3)	0	8 (25.0)	0	9 (23.7)	0
Dizziness	1 (33.3)	0	2 (66.7)	0	6 (18.8)	0	9 (23.7)	0
Alanine aminotransferase increased	0	0	1 (33.3)	0	7 (21.9)	1 (3.1)	8 (21.1)	1 (2.6)
Alopecia	1 (33.3)	0	2 (66.7)	0	5 (15.6)	0	8 (21.1)	0
Pruritus	0	0	1 (33.3)	0	7 (21.9)	0	8 (21.1)	0
Tumor pain	0	0	0	0	8 (25.0)	1 (3.1)	8 (21.1)	1 (2.6)

Duration/dose increases of the fludarabine and cyclophosphamide components of LD chemotherapy were made per the absence of DLTs. An additional day of fludarabine 30 mg/m^2^ dosing was incorporated on day −4 for Group 3 (cyclophosphamide 1,800 mg/m^2^/day on days −3 and −2 and fludarabine 30 mg/m^2^/day on days −5, −4, −3 and −2). Because no hematological DLTs occurred in Group 3, the LD regimen was increased in the expansion group to a higher total dose of cyclophosphamide (3,600 mg/m^2^ administered as 1,800 mg/m^2^ on days −3 and −2), combined with four consecutive days of 30 mg/m^2^ fludarabine. This was deemed scientifically justifiable because high-dose cyclophosphamide LD chemotherapy has been shown to support increased efficacy^9^.

Eighteen deaths were reported due to disease progression (14), AEs (three, two possibly treatment-related AEs) or comorbidities (one). Both treatment-related deaths were among the seven patients in the expansion group who received the highest LD dose regimen. The first was a 77-year-old patient with SS, heavily pre-treated with chemotherapy, who died of aplastic anemia on day 55 of the study. Grade 3 cytopenia developed in this patient from day −5 (LD chemotherapy day 3) and worsened after afami-cel infusion. Bone marrow biopsy on day 38 did not detect myelodysplastic syndrome, cytomegalovirus infection, enrichment of transduced T cells or MAGE-A4 antigen. The second was a 71-year-old patient with ovarian cancer who died of an ischemic cerebrovascular accident on day 17 after a Grade 3 neurotoxicity. Because of these two treatment-related fatalities, the maximum age at screening was reduced to 75 years, and the high-dose cyclophosphamide LD regimen was discontinued. The subsequent 22 patients in the expansion group reverted to the lower cyclophosphamide regimen.

Twenty-one patients (55%) had cytokine release syndrome (CRS)—ten (58%) Grade 1, nine (24%) Grade 2, one (3%) Grade 3 and one (3%) Grade 4; all events resolved (Supplementary Table 4). CRS occurred across all tumor types, with median time to onset of 3 days (range, 1–9) and median duration of 4 days (range, 1–19). Nine of the 19 patients (47%) with Grade 1 or 2 CRS were managed with supportive care. All Grade ≥3 CRS events were in patients with SS, including the patient in the expansion group who died of aplastic anemia.

Two patients (5%) had treatment-emergent, low-grade, early-onset, immune effector cell-associated neurotoxicity syndrome (ICANS)/encephalopathy, which was reversible and lasted 3 days or less: Grade 1 ICANS on day 3 in a patient with NSCLC with known brain metastasis and Grade 2 encephalopathy on day 8 in a patient with ovarian cancer without baseline brain metastasis. Six additional patients had other possible treatment-related neurological AEs, including tremor or headache. Nine expansion group patients had skin rashes possibly related to afami-cel, including three with Grade 3 severity. Skin rashes were typically reversible with supportive care and/or topical corticosteroids. Two patients with SS received a second afami-cel infusion after progressing from partial responses (PRs) achieved with the first afami-cel treatment. No new clinically important TEAEs developed after administration of a second round of LD chemotherapy followed by a second afami-cel infusion compared with their first LD chemotherapy/afami-cel infusion schedules (Supplementary Table 5). Replication-competent lentivirus was not detected in any patient. Based on safety findings, the recommended phase 2 dose of afami-cel was 1.0 × 10^9^ to 10 × 10^9^ transduced cells.

### Clinical activity

**Fig. 2:**
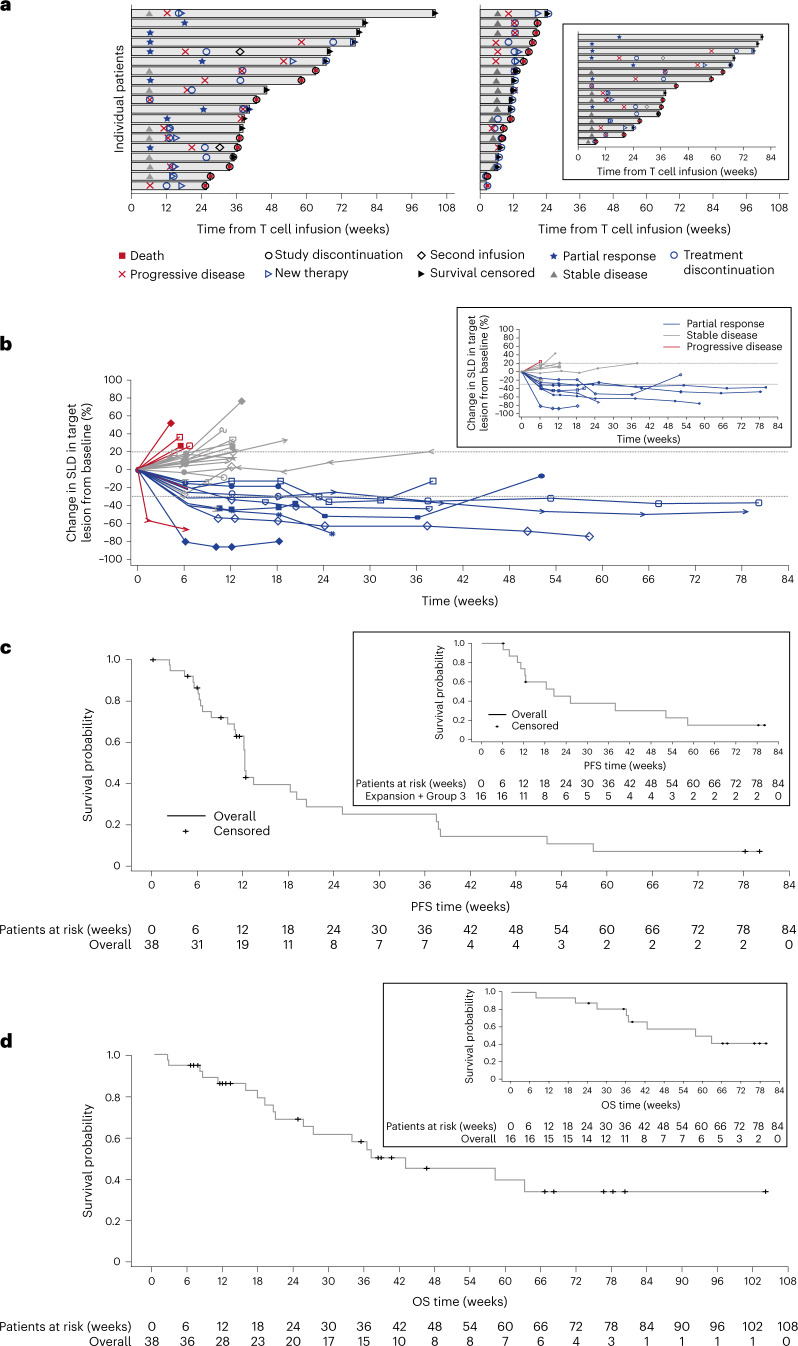
Response and prognostic characteristics of the mITT population and patients with SS. **a**, Swimmer’s plot of patient response over time in the mITT population (*N* = 38). The inset is response over time in patients with SS (*n* = 16). **b**, DoR profiles show the change from baseline in target lesions using RECIST version 1.1 in the overall treatment group after first infusion for responders and non-responders. The inset shows the change from baseline in target lesions in patients with SS. The probability of DoR was 100% (95% CI: 100, 100) at ≥12 weeks and 60% (95% CI: 20.4, 80.5) at ≥24 weeks. Duration of stable disease probability at ≥24 weeks for patients with SS was 50% (95% CI: 22.5%, 75.0%). **c**, Kaplan–Meier curves show PFS in the mITT population. The inset shows the PFS curve for the patients with SS. The median PFS for the mITT population was 12.3 weeks (95% CI: 10.9, 19.1) and 20.4 weeks (95% CI: 10.0, 52.1) for the patients with SS. PFS events in the mITT population included 25 events (65.8%) of PD and four events (10.5%) of death. PFS probability was 60% (95% CI: 44.8, 76.6) at 12 weeks and 30% (95% CI: 14.1, 45.2) at 24 weeks. PFS probability in the patients with SS was 70% (95% CI: 0.44, 0.89) at 12 weeks and 50% (95% CI: 0.19, 0.68) at 24 weeks. **d**, Kaplan–Meier curves show OS in the mITT population. The inset shows the OS for patients with SS. The median OS for the mITT population was 42.9 weeks (95% CI: 20.7, not reached) and 58.1 weeks (95% CI: 36.3, not reached) for patients with SS. OS probability was 90% (95% CI: 69.8, 94.0) at 12 weeks and 70% (95% CI: 49.6, 82.1) at 24 weeks. OS probability in patients with SS was 90% (95% CI: 0.63, 0.99) at 12 weeks and remained 90% (95% CI: 0.59, 0.97) at 24 weeks.

**Extended Data Fig. 3:**
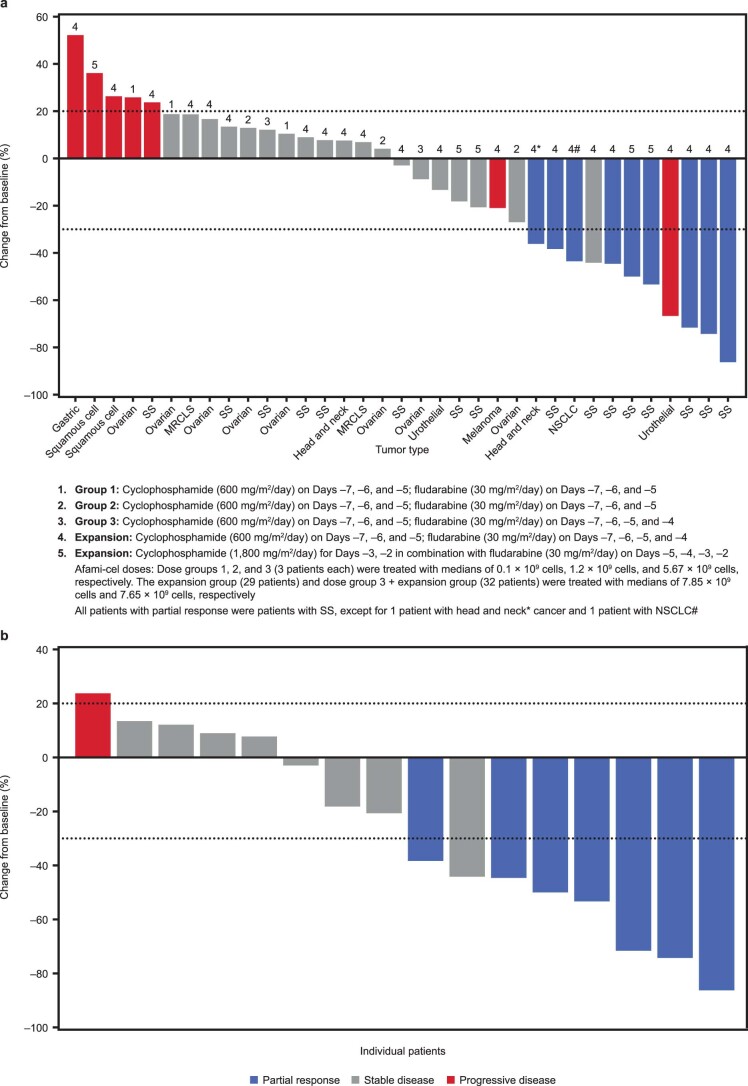
Maximal change in target lesion from baseline using RECIST v1.1 (modified intent-to-treat population; *N* = 38). (**a**) Maximal change in target lesion from baseline in the modified intent-to-treat population. Data for three patients were not available as of data cut-off on 1 September 2020. Each bar represents an individual patient. Nine patients responded to first infusion as determined by RECIST v1.1 criteria. All nine responders (23.7%) had a partial response with an overall response rate of 23.7% (95% CI: 11.4, 40.2). (**b**) Synovial sarcoma subgroup: Maximal change in target lesion from baseline in the synovial sarcoma subgroup. Each bar represents an individual patient. Seven of the nine responders were in the synovial sarcoma subgroup (*n* = 16) with a best overall response of partial response and an overall response rate of 43.8% (95% CI: 19.8, 70.1). Horizontal reference lines at -30% and 20%. CI, confidence interval; MRCLS, myxoid/round cell liposarcoma; NSCLS, non-small cell lung cancer; RECIST, Response Evaluation Criteria in Solid Tumors; SS, synovial sarcoma.

In the mITT population, ORR was 24% (95% confidence interval (CI): 11.4, 40.2; 9/38 patients) (Fig. 2a and Extended Data Fig. 3a). All nine patients with BOR of PR were treated in the Group 3/expansion cohort (highest T cell infusion group—seven SS, one HNSCC and one NSCLC of squamous histology) (Supplementary Table 6). The change in sum of longest diameter (SLD) of target lesions over time is shown in Fig. 2b. All responders had tumor MAGE-A4 H-scores of >200, except one with HNSCC. The disease control rate (DCR) (percentage of patients with objective response or SD) was 74% (nine PR and 19 SD), including five of six patients with ovarian cancer in Groups 1 and 2. The median TTR was 6.4 weeks (95% CI: 6.1, 24.1), and median DoR was 25.6 weeks (95% CI: 12.3, not reached). The median PFS was 12.3 weeks (95% CI: 10.9, 19.1), and median OS was 42.9 weeks (95% CI: 20.7, not reached) (Fig. 2c,d). CRS was more frequent in responders. CRS occurred in 89% of patients with PR, 47% with SD and 43% with progressive disease (PD). Objective responses were not observed in the two patients with SS who received a second infusion of afami-cel (Fig. 2a).

In the 16 heavily pre-treated metastatic SS patients, ORR was 44% (95% CI: 19.8, 70.1) (Fig. 2a, inset, and Extended Data Fig. 3b), and DCR was 94% (seven PR and eight SD). The change in SLD of the target lesions over time for patients with SS is shown in Fig. 2b (inset). Median TTR was 6.4 weeks (95% CI: 6.1, 18.1), and median DoR was 28.1 weeks (95% CI: 12.3, not reached). All seven responding patients had high tumor MAGE-A4 H-scores >214 and were treated with afami-cel doses >9.5 × 10^9^, except one patient receiving 4.5 × 10^9^ cells. Median PFS was 20.4 weeks (95% CI: 10.0, 52.1) (Fig. 2c, inset), and median OS was 58.1 weeks (95% CI: 36.3, not reached) (Fig. 2d, inset), with 81%, 44% and 13% alive at 6 months, 12 months and 18 months, respectively. Three patients were progression free for >12 months.

**Extended Data Fig. 4:**
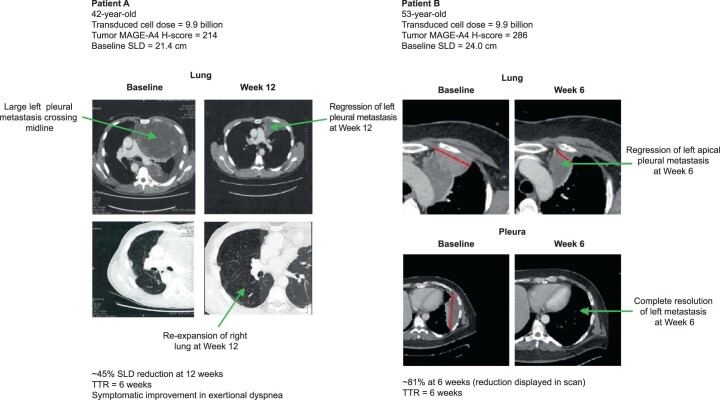
Afami-cel therapy leads to regression of large pleural-based thoracic metastasis in synovial sarcoma. In Patient A, afami-cel was associated with reductions in metastatic disease in hemithorax, including regression of a large left lung pleural metastasis that crossed the midline at baseline (Patient A, upper right and upper left panels, respectively) and re-expansion of the right lung, as shown on a computed tomography scan at Week 12 post−afami-cel (Patient A, lower right panel, arrow), associated with patient-reported improvement in exertional dyspnea. In Patient B, afami-cel was associated with overall reduction in left lung pleural metastases including complete resolution of one pleural metastasis (Patient B, lower right panel, arrow). BOR, best overall response; MAGE-A4, melanoma-associated antigen A4; PR, partial response; SLD, sum of longest diameter; TTR, time to response.

Because disease-specific mortality in SS is mediated by progression of pulmonary and pleural metastases, radiologically confirmed durable regression of intrathoracic lesions after afami-cel could suggest clinical benefit. For example, pre-infusion and post-infusion cross-sectional thoracic contrast computed tomography scans in two patients with SS (each with MAGE-A4 H-scores >200, large baseline tumor burdens and treated with high afami-cel doses) confirmed the in vivo potency and anti-tumor activity of afami-cel (Patients A and B; Extended Data Fig. 4 and Supplementary Table 5). Both patients had PRs with TTR at week 6 and SLD reductions of −45% (at 12 weeks) and −81% (at 6 weeks), respectively, principally in pleural metastasis target lesions (Extended Data Fig. 4 and Supplementary Table 5). In Patient A, afami-cel was associated with reductions in metastatic disease in hemithorax, including regression of a large left lung pleural metastasis that crossed the midline at baseline and re-expansion of the right lung, as shown on a computed tomography scan at week 12 after afami-cel (Extended Data Fig. 4), associated with patient-reported improvement in exertional dyspnea. In Patient B, afami-cel was associated with overall reduction in left lung pleural metastases, including complete resolution of one pleural metastasis (Extended Data Fig. 4).

Patients A and B were administered a second T cell infusion (manufactured from surplus cell material collected at the initial leukapheresis) after disease progression from their first afami-cel infusions. Neither of the second infusions led to a response. Baseline tumor biopsy before the second infusion in Patient A showed markedly lower MAGE-A4 expression compared with a tumor biopsy before the first infusion (H-score, 37 versus 214, respectively). For Patient B, MAGE-A4 expression was high in biopsies taken before the first and second infusions (Supplementary Table 5).

### Translational analyses

Exploratory analyses aimed to evaluate peripheral and tumor profiles relating afami-cel, including its circulating persistence and pharmacodynamic immune markers, and the tumor microenvironment before infusion and after infusion. This included immunophenotyping and cytotoxicity of afami-cel manufactured product (MP), serum cytokine profiling and spatial protein and gene expression profiling of the tumor microenvironment.

#### Exploratory peripheral analyses

**Extended Data Fig. 5:**
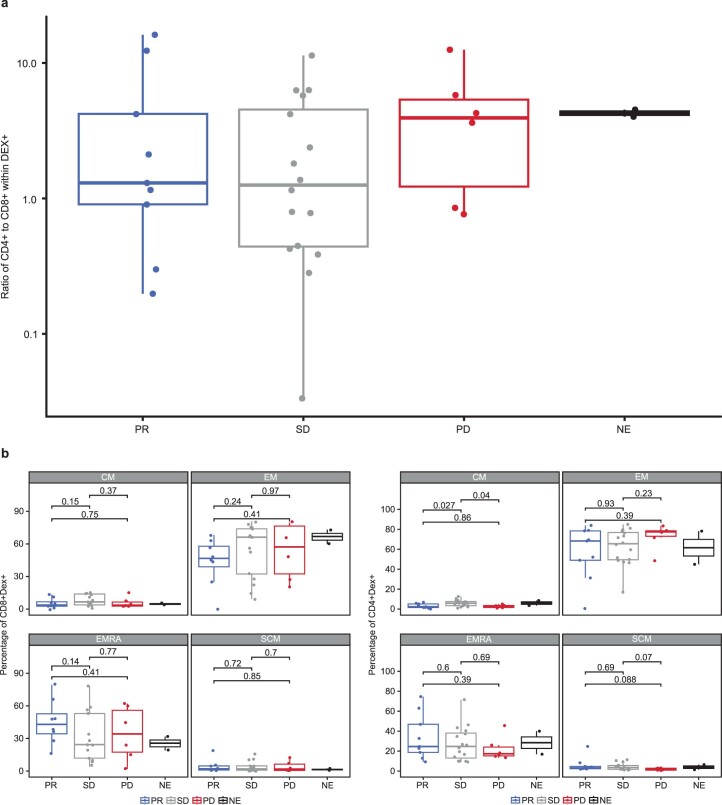
Ratio of transduced CD4^+^ to CD8^+^ cells and memory sub-populations in afami-cel manufactured product. (**a**) Ratio of CD4^+^ to CD8^+^ cell subsets within the transduced cells infused, derived from immunophenotyping: Ratios greater than 1 denote a CD4 bias in the transduced cells. Horizontal lines denote median values. Samples are grouped and color coded by BOR resulting from the infusion (blue PR, gray SD, red PD, black NE). Ratio of PR range 0.19–16.16 (median 1.30, *n* = 9) vs. SD range 0.03–11.58 (median 1.26, *n* = 16) vs. PD range 0.76–12.52 (median 3.94, *n* = 6). (**b**) Memory subset distribution by immunophenotyping within the transduced cells infused: The proportion of live single transduced afami-cel within the respective memory subsets is indicated by box plots for CD8^+^ afami-cel on the left and for CD4^+^ on the right. Samples are grouped and color coded by BOR resulting from the infusion (blue PR, gray SD, red PD, black NE). CM = central memory, CD45RA^-^CCR7^+^; EM = effector memory, CD45RA^-^CCR7^-^; EMRA = effector memory RA^+^, CD45RA^+^ CCR7^-^; SCM = stem cell memory, CD45RA^+^ CCR7^+^. Two-sided paired Wilcoxon test *P* values are shown linking compared cell types. Box plots depict median as horizontal lines within boxes, with box bounds as the first and third quartiles. Dots represent individual data points. Lower whisker is the minimum value of the data within 1.5 times the interquartile range below the 25^th^ percentile. Upper whisker is the maximum value of the data within 1.5 times the interquartile range above the 75^th^ percentile. BOR, best overall response; DEX, dextramer; NE, not evaluable; PD, progressive disease; PR, partial response; SD, stable disease.

Afami-cel MP showed in vitro cytotoxic activity in all batches before dosing. In vitro killing of MAGE-A4^+^ tumor cells by CD8^+^ afami-cel was significantly greater compared with CD4^+^ afami-cel (at 72 hours: median, 79.9% (range, 33.5−97.6%; *n* = 37) versus median, 6.6% (range, −8.7% to 52.9%; *n* = 35), respectively; *P* = 5.8 × 10^−11^ (paired Wilcoxon test)). The immunophenotype profile, examined for 33 patient MP samples showed transduced CD4^+^ to CD8^+^ cell ratios between 16.16 (CD4-biased) and 0.03 (CD8-biased) (median, 1.81), with no significant association with clinical response (Extended Data Fig. 5a). Infused MPs had CD4^+^ and CD8^+^ afami-cel predominantly resembling effector memory cells (T_EM_; CCR7^−^CD45RA^−^) (median, 69.5% and 56.5%, respectively) and terminally differentiated effector memory cells (T_EMRA_; CCR7^−^CD45RA^+^) (median, 23.3% and 36%, respectively). Stem cell memory (T_SCM_; CD45RA^+^CCR7^+^) and central memory (T_CM_; CD45RA^−^CCR7^+^) subsets were less frequent in the infused MPs (generally <10% and <15%, respectively). No association was observed between prevalence of these subsets and clinical response (Extended Data Fig. 5b).

Persistence of afami-cel was detected in all post-infusion blood samples up to 18 months after treatment. Peak afami-cel persistence was reached in most patients within the first 7 days after infusion. Integration site analysis evaluated clonality status. In five patients with persistence >1% of peripheral blood mononuclear cells (PBMCs) 1 year after infusion, afami-cel showed a high level of polyclonality and absence of clonal dominance (Supplementary Table 7).

To assess whether long-term persisting afami-cel retains its functional cytolytic capacity, a sample was taken ~9 months after afami-cel infusion from a patient with SS with durable PR for 28 weeks who eventually progressed. CD8^+^ afami-cel retained its functional capacity for HLA-directed tumor cell lysis in vitro (75% of targets killed in 72 hours, 93% killed in 125 hours), suggesting that loss of response in this patient was not associated with loss of CD8^+^ T cell function.

**Extended Data Fig. 6:**
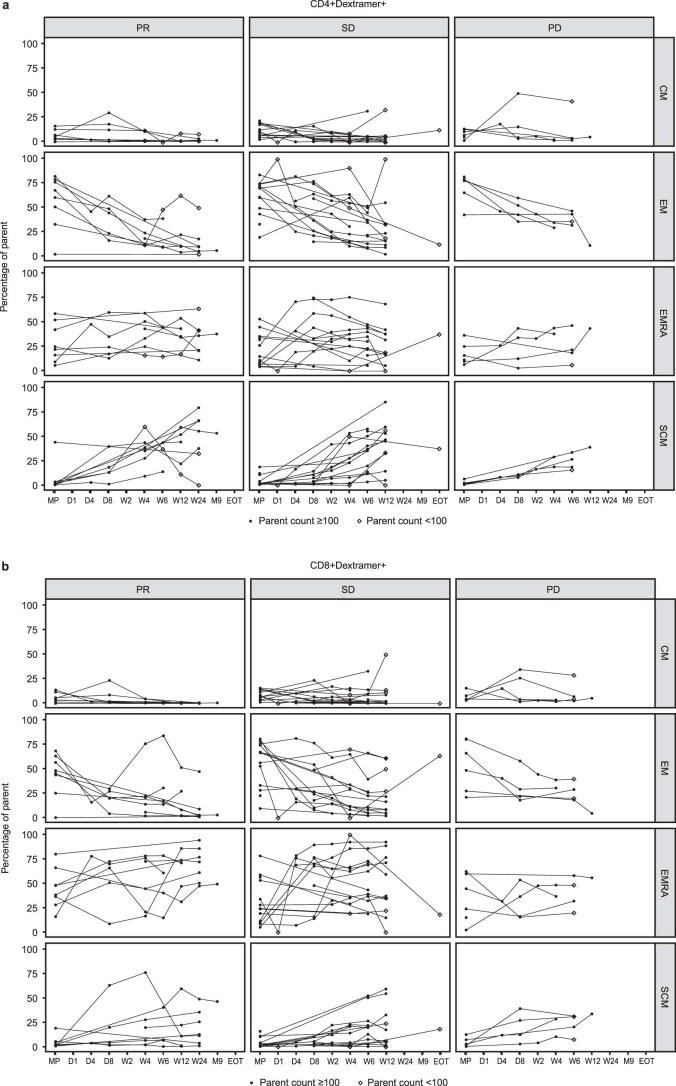
Longitudinal immunophenotyping of memory subtypes within circulating afami-cel. Percentage of parent population (**a**) CD4^+^ Dextramer^+^ and (**b**) CD8^+^ Dextramer^+^ within the respective sub-population flow gate in MP (manufactured product) and peripheral blood mononuclear cell samples at post-infusion timepoints (D, day; W, week; M, month, EOT, end of treatment). CM (central memory CD45RA^-^CCR7^+^); EM (effector memory CD45RA^-^CCR7^-^); EMRA (effector memory RA^+^, CD45RA^+^ CCR7^-^); SCM (stem cell memory, CD45RA^+^ CCR7^+^). Data are grouped by best overall response resulting from the infusion. PR (partial response), *n* = 9 patients; SD (stable disease), *n* = 17 patients; PD (progressive disease), *n* = 6 patients.

Longitudinal changes in memory subset populations in the transduced CD4^+^ and CD8^+^ afami-cel pool were profiled in post-infusion PBMC samples. A gradual increase in the proportion of circulating cells with a T_SCM_ phenotype and sustained presence of T_EMRA_ cell types was observed (Extended Data Fig. 6).

**Fig. 3:**
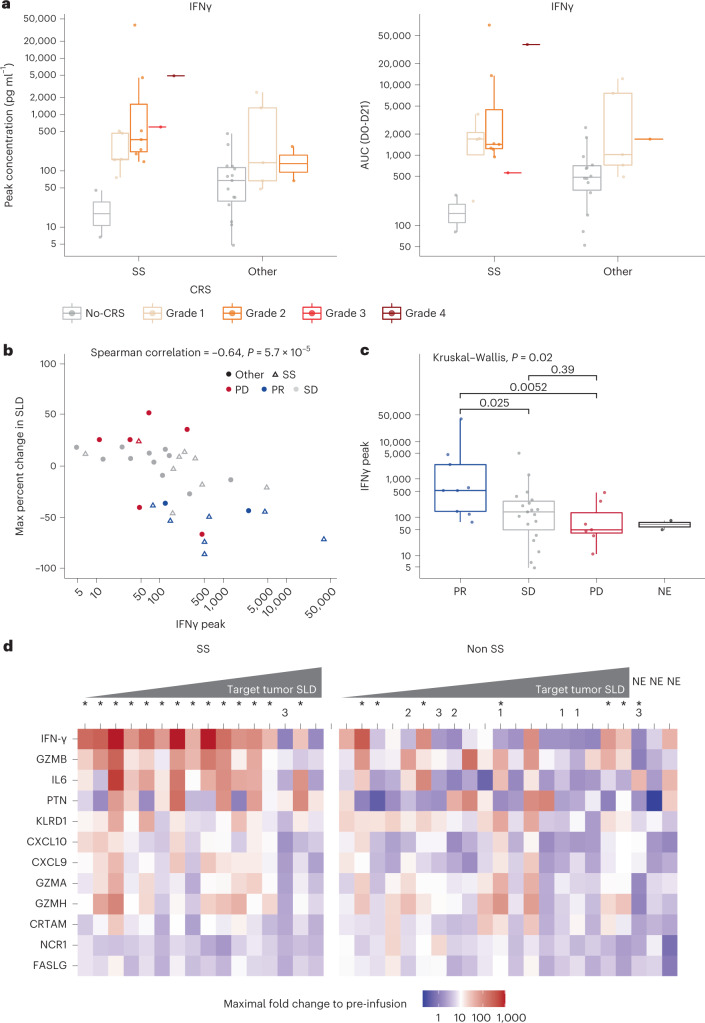
Serum IFNγ patient profiles and associations between peak and AUC concentrations of IFNγ levels and anti-tumor response. **a**, Comparison of serum levels in patients with SS (no CRS *n* = 2, Grade 1 *n* = 5, Grade 2 *n* = 7, Grade 3 *n* = 1, Grade 4 *n* = 1) and those with other indications (no CRS *n* = 15, Grade 1 *n* = 5, Grade 2 *n* = 2) across CRS groups. Peak IFNγ levels (pg ml^−1^) were significantly greater in patients with CRS (all grades, *n* = 21) compared with non-CRS (*n* = 17) (median, 270.8 pg ml^−1^ and 47.7 pg ml^−1^, respectively; *P* = 0.00012). Calculated IFNγ AUC from days 0 to 21 was significantly greater in patients with CRS compared with non-CRS (median, 1,455.0 pg ml^−1^ and 467.0 pg ml^−1^, respectively; *P* = 0.00061) (Wilcoxon rank-sum test, two-sided). **b**, Serum IFNγ concentration (pg ml^−1^) measured across sample sets collected on days 0–21 after infusion. Magnitude of maximum percent change in SLD was positively correlated with reduction in target tumor lesion (Spearmanʼs *r* = −0.64; *P* = 0.000057). **c**, Peak serum IFNγ levels were significantly greater in patients with best overall responses of PR (*n* = 9) compared with PD (*n* = 7; *P* = 0.0052) and SD (*n* = 19; *P* = 0.025) (Kruskal–Wallis test, two-sided). **d**, Patients ranked by best percent change in SLD. Non-SS subset included three NE patients. The asterisk in superscript (^*^) indicates CRS; number indicates dose-escalation cohort. AUC, area under the curve; CRTAM, class I-restricted T cell-associated molecule; CXCL, chemokine (C-X-C motif) ligand; FASLG, FAS ligand; IQR, interquartile range; KLRD1, killer cell lectin like receptor D1; NCR1, natural cytotoxicity triggering receptor 1; PTN, pleiotrophin. Box plots depict median as horizontal lines within boxes, with box bounds as the first and third quartiles. Dots represent individual data points. Lower whiskers are minimum values within 1.5 times the IQR below the 25th percentile. Upper whiskers are maximum values within 1.5 times the IQR above the 75th percentile.

**Extended Data Fig. 7:**
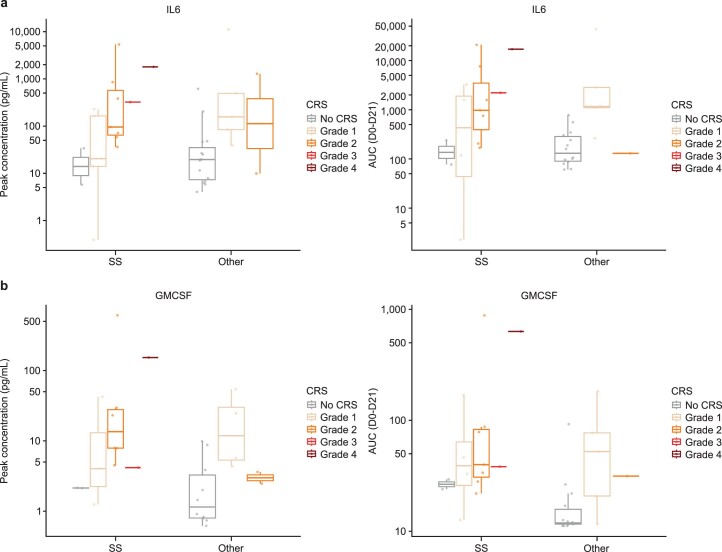
Serum cytokine profiles associated with cytokine release syndrome. Peak IL-6 levels pg ml^-1^ (**a**, left panel), calculated IL-6 AUC Day 0–21 (**a**, right panel), peak GMCSF levels pg ml^-1^ (**b**, left panel) and calculated GMCSF AUC Day 0–21 (**b**, right panel) were significantly greater in patients with CRS (all grades, *n* = 21) compared with non-CRS (*n* = 17) in the mITT population (Supplementary Table 8). Box plots show comparison of serum levels in SS patients (no CRS *n* = 2, Grade 1 *n* = 5, Grade 2 *n* = 7, Grade 3 *n* = 1, Grade 4 *n* = 1) and those with other indications (no CRS *n* = 15, Grade 1 *n* = 5, Grade 2 *n* = 2) across CRS groups. Box plots depict median as horizontal lines within boxes, with box bounds as the first and third quartiles. Dots represent individual data points. Lower whisker is the minimum value of the data within 1.5 times the interquartile range below the 25^th^ percentile. Upper whisker is the maximum value of the data within 1.5 times the interquartile range above the 75^th^ percentile. AUC, area under the curve; CRS, cytokine release syndrome; GMCSF, granulocyte-macrophage colony-stimulating factor; IL, interleukin; SS, synovial sarcoma.

A panel of 22 serum cytokines was simultaneously assessed in mITT patient samples collected before infusion and after infusion to understand potential mechanisms of toxicity and efficacy. A transient post-infusion increase was evident for most serum cytokines across all patients, with the largest increases in interferon γ (IFNγ) levels. Serum IFNγ (Fig. 3a), interleukin 6 (IL-6) (Extended Data Fig. 7a) and granulocyte–macrophage colony-stimulating factor (GMCSF) (Extended Data Fig. 7b) levels were relatively greater in patients with Grade ≥3 CRS versus Grade ≤2 CRS and without CRS (Supplementary Table 8).

**Extended Data Fig. 8:**
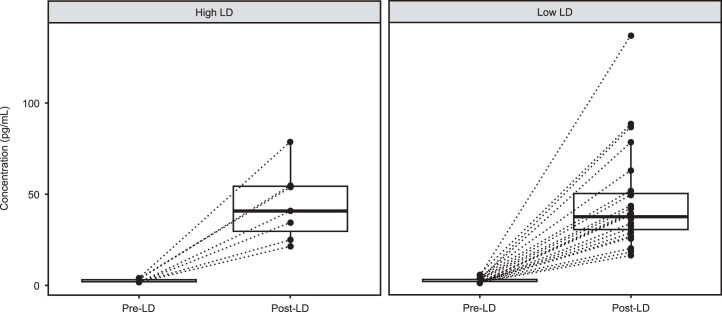
IL-15 serum concentrations in patients treated with high dose (3600 mg/m^2^) or low dose (1800 mg/m^2^) lymphodepleting chemotherapy pre- and post-lymphodepletion. Serum IL-15 levels pg ml^-1^ were measured pre- and post-LD in Group 3/Expansion patients treated with ‘high’ LD (*n* = 7) or ‘low’ LD (*n* = 24*) regimen (*excludes one patient with an unevaluable baseline sample). Box plots depict median as horizontal lines within boxes, with box bounds as the first and third quartiles. Dots represent individual data points. Lower whisker is the minimum value of the data within 1.5 times the interquartile range below the 25^th^ percentile. Upper whisker is the maximum value of the data within 1.5 times the interquartile range above the 75^th^ percentile. IL, interleukin; LD, lymphodepletion.

**Extended Data Fig. 9:**
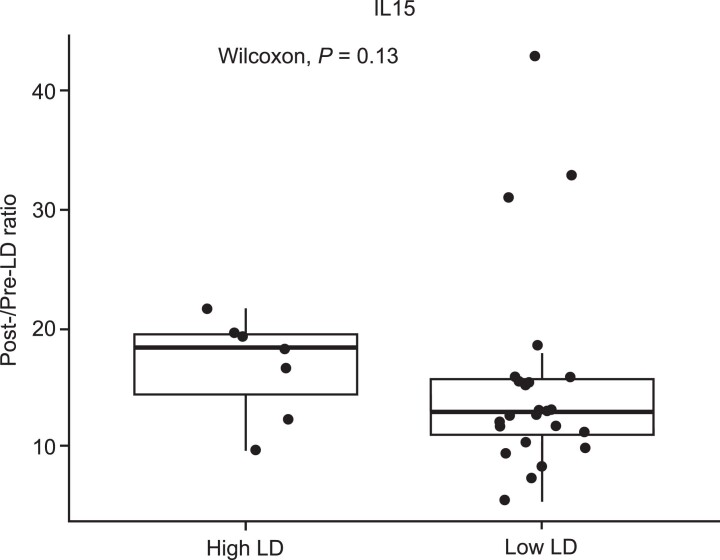
Post-/pre-lymphodepleting chemotherapy serum IL-15 ratio in patients treated with high dose (3600 mg/m^2^) or low dose (1800 mg/m^2^) lymphodepleting chemotherapy. Serum IL-15 concentration increased in post-LD versus pre-LD samples, and there was no difference in IL-15 concentrations between the two LD regimens in patients treated in Group 3/Expansion (median of ‘high’ LD 18.25 (*n* = 7), median of ‘low’ LD 12.87 (*n* = 24); *P* = 0.13, Two-sided Wilcoxon test). Box plots depict median as horizontal lines within boxes, with box bounds as the first and third quartiles. Dots represent individual data points. Lower whisker is the minimum value of the data within 1.5 times the interquartile range below the 25^th^ percentile. Upper whisker is the maximum value of the data within 1.5 times the interquartile range above the 75^th^ percentile. IL, interleukin; LD, lymphodepletion.

Analyses of serum cytokine relationships with clinical response showed a significant correlation between peak serum IFNγ levels and tumor reduction (Fig. 3b). Peak serum IFNγ levels were significantly greater in responders than non-responders (Fig. 3c). No significant difference was noted among PD, SD and not evaluable (NE) patient subsets. As anticipated for the mechanism of action for LD chemotherapy^10^, serum IL-15 concentrations increased in post-LD versus pre-LD samples, but there was no difference in serum IL-15 concentrations between the two LD chemotherapy regimens. The median post-LD versus pre-LD ratio of IL-15 was 18.25 for the high LD group and 12.87 for the low LD group (*P* = 0.13 (two-sided Wilcoxon test)) (Extended Data Figs. 8 and 9).

Further pharmacodynamic analyses of afami-cel were supported by the evaluation of 92 immuno-oncology-related serum proteins using Olink technology. Markers with the greatest afami-cel infusion-related changes included IFNγ and related markers. After afami-cel, fold increases in serum IFNγ levels relative to baseline were associated with anti-tumor response; this was more evident in SS than other tumors (Fig. 3d). Overall, peak levels of 14 serum markers after treatment correlated with tumor reduction, and serum IFNγ levels showed the largest positive correlation (Supplementary Table 9).

#### Exploratory tumor analyses

**Extended Data Fig. 10:**
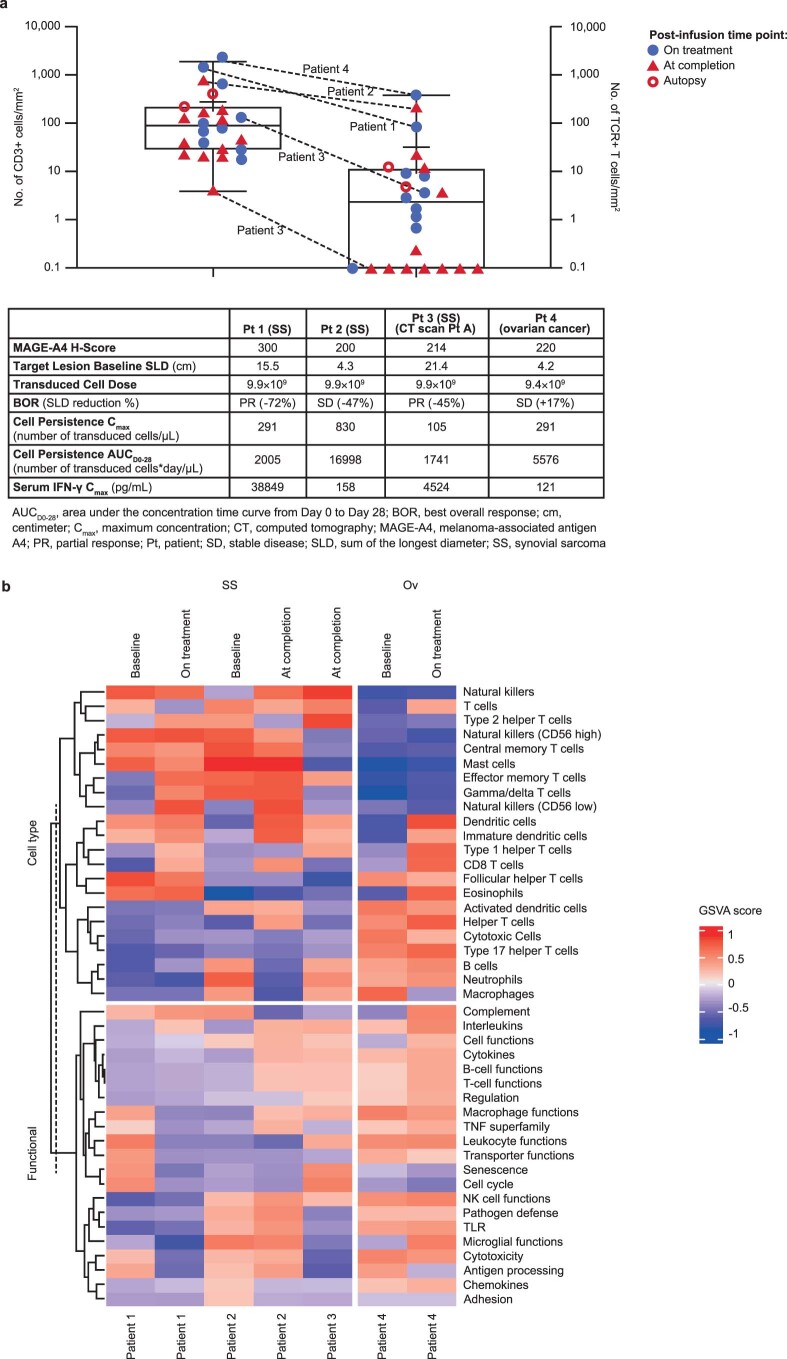
Detection of afami-cel and characterization of phenotypes in patient tumor biopsies. (**a**) Quantification of CD3^+^ T cells and afami-cel in post-infusion tumor biopsies from dose expansion: Four patients with contrasting profiles were prioritized for multiplex immunofluorescence analyses (summarized in Fig. 4a). Patient 1, SS, on-treatment biopsy taken 8 weeks post-infusion; Patient 2, SS, at completion biopsy taken 17 weeks post-infusion; Patient 3, SS, on-treatment and at-completion biopsies taken 7 and 25 weeks post-infusion, respectively; Patient 4, Ov tumor, on-treatment biopsy taken 6 weeks post-infusion. Table summarizes key baseline tumor characteristics, afami-cel dose, post-infusion pharmacokinetic-pharmacodynamic findings, and BOR status for these four patients. Patients 1-3 were patients with SS; Patient 4 was a patient with Ov cancer. Box plots depict median as horizontal lines within boxes, with box bounds as the first and third quartiles. Dots represent individual data points. Lower whisker is the minimum value of the data within 1.5 times the interquartile range below the 25^th^ percentile. Upper whisker is the maximum value of the data within 1.5 times the interquartile range above the 75^th^ percentile. (**b**) Heatmap of GSVA scores of cell type specific and immune response categories gene lists in baseline and post-infusion biopsies from Patients 1‒4. AUC, area under the curve; BOR, best overall response; C_max_, maximum serum concentration; CT, computed tomography; GSVA, gene set variation analysis; IFN, interferon; MAGE-A4, melanoma-associated antigen A4; NK, natural killer; Ov, ovarian; PR, partial response; Pt, patient; SD, stable disease; SLD, sum of longest diameter; SS, synovial sarcoma; TCR, T-cell receptor; TLR, toll-like receptor; TNF, tumor necrosis factor.

Infiltration of non-transduced T cells and afami-cel was evaluated in 24 post-infusion biopsies from 16 patients treated in the expansion phase (Extended Data Fig. 10a). CD3^+^ cells were detected in all samples (range, 4–1,891 CD3^+^ cells per mm^2^ (median, 90)), with afami-cel evident in 67% of samples (16/24; range, 0–385 transduced T cells per mm^2^ (median, 2.3)), including biopsies obtained at study completion and autopsy. Of biopsies without afami-cel detection, seven of eight were taken at study completion. Biopsies were taken from 6 weeks to 93 weeks (median, 67) after infusion and compared with the earlier biopsies taken from 6 weeks to 67 weeks (median, 12) from samples with afami-cel detected; MAGE-A4 expression was similar in both.

**Fig. 4:**
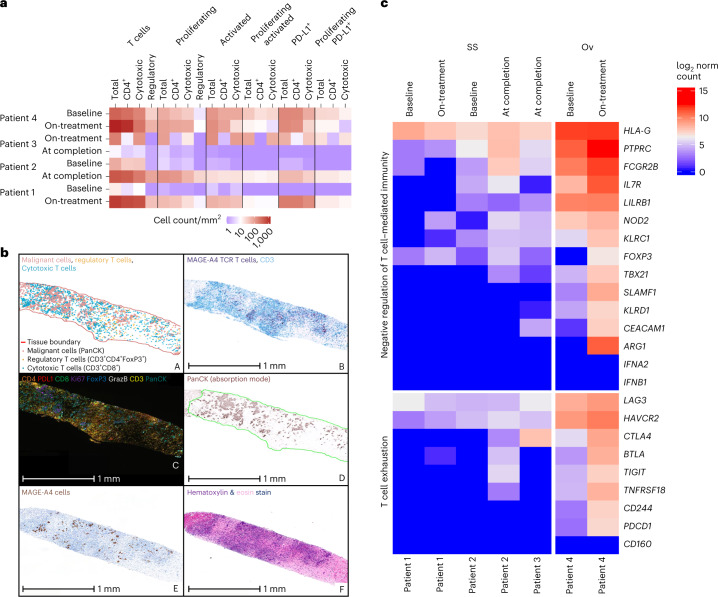
Detection of afami-cel and T cell phenotypes in patient tumor biopsies. **a**, Digital image quantification of T cell markers (total CD3^+^), CD4 (CD3^+^CD4^+^), cytotoxic (CD3^+^CD8^+^), regulatory (CD3^+^CD4^+^FoxP3^+^) and co-staining for phenotypes (proliferating, Ki67^+^; activated, GrazB^+^; PD-L1^+^) and combination of these phenotypes for the four patients referenced in Extended Data Fig. 10a. Baseline biopsies were taken 2–5 weeks before afami-cel infusion. **b**, A multiparametric analysis of T cell infiltration in tumor biopsies using IHC, MAGE-A4 SPEAR RNAscope (Advanced Cell Diagnostics) and multiplex immunofluorescence. Images of post-infusion biopsy (liver) from Patient 4: A, spatial plot generated using spatial analysis module in HALO (Indica Labs) showing malignant cells (PanCK^+^ (red), regulatory (CD3^+^CD4^+^FoxP3^+^) T cells (yellow) and cytotoxic (CD3^+^CD8^+^) T cells (blue/cyan)). Scale bar is not applicable as this is not a raw image; B, CD3 IHC/SPEAR^+^ T cell RNAscope duplex (CD3 IHC staining (blue); MAGE-A4 SPEAR T cell staining (purple)); C, Ultivue 8-plex multiplex dataset showing CD4 (orange), PD-L1 (red), CD8 (green), Ki67 (purple), FoxP3 (blue), GrazB (white), CD3 (yellow) and PanCK (teal); D, PanCK immunofluorescence staining from Ultivue 8-plex panel (displayed in absorption mode in HALO for clarity); E, MAGE-A4 IHC stain (DAB (brown)); and F, hematoxylin (purple) and eosin (pink) stain. **c**, Heat map of log_2_-transformed normalized counts of genes associated with ‘T cell exhaustion’ and ‘Negative regulation of T cell-mediated immunity’ in baseline and post-infusion biopsies from Patients 1–4. Patients 1–3 were patients with SS; Patient 4 was a patient with ovarian cancer. DAB, 3,3′-diaminobenzidine; FoxP3, forkhead box protein 3; GrazB, granzyme B; Ov, ovarian; PanCK, pancytokeratin; PD-L1, programmed death ligand 1.

To examine the tumor microenvironment for patients with a differential response to afami-cel after receiving similar (high) cell doses (9.0–10 × 10^9^ transduced T cells), a sample set including on-study and at-completion biopsies from four patients across two tumor types (Patients 1, 2 and 3 had SS and Patient 4 had ovarian cancer; Extended Data Fig. 10a) was prioritized for multiplex immunofluorescence analyses. Compared with baseline, relatively greater intra-tumoral detection of proliferating (Ki67^+^) and/or activated (granzyme B^+^ and PD-L1^+^) T cells (CD4, CD8 and ‘regulatory’) was evident after afami-cel in on-study (Patient 1) and at-completion/withdrawal (Patient 2) biopsies, in line with afami-cel detection. For Patient 3, a relatively lower intra-tumoral detection of proliferating and/or activated T cells was observed at completion compared with the on-study biopsy, consistent with low/negligible afami-cel detection (Fig. 4a). At baseline, an ovarian tumor biopsy (Patient 4) had a relatively higher presence of T cell phenotypes compared with SS tumor biopsies (Patients 1 and 2). Post-infusion ovarian tumor biopsy (Patient 4) also showed a high level of afami-cel infiltrates (Fig. 4b, panel B) in areas that corresponded spatially with those showing the presence of cytotoxic and regulatory T cells near malignant cells (Fig. 4b, panels A and C).

The absence of a clinical response in Patient 4, despite high-dose afami-cel (9.4 × 10^9^ transduced cells) and evidence of high levels of intra-tumoral immune cell infiltration (including afami-cel), could possibly be explained by the presence of immunosuppressive markers.

NanoString nCounter analyses showed that intra-tumoral gene expression levels encoding T cell exhaustion markers (that is, LAG-3, TIGIT, CTLA-4 and PD-1) and immunosuppressive markers, including arginase (for which serum levels were negatively correlated with clinical response), were relatively higher in biopsy from Patient 4 (on-treatment greater than baseline) compared with SS samples (Fig. 4c). Furthermore, a gene set variation analysis comparing scores for PanCancer Immune Profiling Panel categories also indicated differences between Patient 4 (ovarian cancer) and patients with SS. The former showed negative enrichment for natural killer cells and central and effector memory T cells and positive enrichment for cytotoxic cells and type 17 helper T cells, contrasting with SS samples (Extended Data Fig. 10b).

## Discussion

In this phase 1 trial, we evaluated the safety, clinical activity and translational effects of afami-cel in HLA-A*02^+^ patients with MAGE-A4-expressing solid tumors. Prolonged cytopenia, CRS and neurotoxicity were three TEAEs of special interest. The prolonged cytopenia-related fatality in a patient with SS caused by aplastic anemia might have been caused by the higher LD dose of 3,600 mg/m^2^ of cyclophosphamide. Because this patient also had two sequential high-grade CRS events, it cannot be excluded that there was a contributory systemic inflammatory component to this Grade 5 event. Reassuringly, there was no evidence of an off-tumor, on-target effect observed in the bone marrow because MAGE-A4 expression was absent. However, although 45% of patients had at least one prolonged Grade ≥3 cytopenia, the incidence of clinical sequelae, including systemic infections, was low. Overall, the lower-dose cyclophosphamide LD regimen was associated with a favorable hematological toxicity profile and a similar serum IL-15 profile compared to patients treated with the higher-dose cyclophosphamide LD regimen. Given the overall favorable hematological safety profile of fludarabine 30 mg/m^2^ × 4 days and cyclophosphamide 600 mg/m^2^ × 3 days LD chemotherapy and its ability to support afami-cel anti-tumor activity across different indications, with no discernable difference in serum IL-15 levels compared to the higher cyclophosphamide dose schedule, the lower LD chemotherapy regimen was selected for the registration-directed phase 2 SPEARHEAD-1 trial (NCT04044768).

Afami-cel-related CRS occurred in 55% of all patients and was typically low-grade, early-onset, post-infusion and reversible in all cases with the administration of anti-IL-6(R) monoclonal antibody treatment when indicated. Consistent with the known monocyte–macrophage-centric pathophysiology of CRS, elevations in serum cytokines (including IFNγ, IL-6 and GMCSF) were associated with increasing CRS grades. The overall incidence and severity of ICANS/encephalopathy was low, with no reported cases in patients with SS. Although additional neurological AEs were reported across tumor types, there was no consistent pattern to suggest a definite causal relationship with afami-cel. The safety findings reported here are consistent with those observed in patients with cancer undergoing LD chemotherapy and cellular therapy, including treatment with chimeric antigen receptor T cells or NY-ESO-1 T cells^11–13^.

Patients treated in the trial had nine different cancers with heterogenous MAGE-A4 expression, and most patients with therapeutic responses had high H-scores. The ORR of 24% in the overall population was primarily due to clinical activity observed in SS. Responses were limited in non-sarcoma cancers, perhaps due to small numbers of patients with specific cancer types or lower MAGE-A4 expression relative to SS. Although the number of patients with SS was small, the emergent ORR of 44%, median PFS of 20.4 weeks and median OS of 58.1 weeks are better than the historical low ORRs reported for current standard-of-care therapies, including pazopanib and trabectedin, used in the post-first-line metastatic setting^14,15^. SS is sensitive to alkylators, and the use of cyclophosphamide in the LD regimen is a confounder in the interpretation of the response rate in SS.

Preliminary evaluation of pharmacokinetic and pharmacodynamic relationships associated with afami-cel dose escalation used peak cell persistence and peak serum IFNγ levels as prototypical pharmacokineticand pharmacodynamic markers, respectively. When considering the subset of patients with ovarian cancer across all dosing groups, increases in afami-cel doses were associated with progressively increasing peak cell persistence and peak IFNγ levels (Supplementary Table 10). Although cyclophosphamide-containing LD chemotherapy, or persisting therapeutic effects of bridging therapy, may have contributed to disease control in ovarian cancer and SS, the independent therapeutic activity of afami-cel in responding patients is corroborated by cell pharmacokinetic and serum pharmacodynamic findings. The durable reduction in SLD for several months in some patients with SS after one afami-cel infusion suggests that the anti-tumor effect of afami-cel lasts longer than would be expected after one cycle of alkylating-agent-containing LD chemotherapy. Although some patients with PD had rapidly decreasing levels of afami-cel persistence, some maintained high levels of persistence, suggesting that tumor-intrinsic factors, possibly immuno-editing mechanisms (for example, loss of MAGE-A4 or HLA expression), or potential immunosuppressive factors (such as arginase-1), might contribute to the development of afami-cel resistance even if afami-cel cytolytic activity can be maintained in vivo.

The serum cytokine response profile indicated an IFNγ-driven mechanism of action as well as emerging biomarkers of anti-tumor response. Tumor analyses supported the co-localization of afami-cel with cancer cells and resident immune cells in tumors, with evidence of activated and proliferative intra-tumoral T cell states and adaptive immune responses. Because the ongoing phase 2 SPEARHEAD-1 trial will have dosed ~100 patients with SS and MRCLS with afami-cel at completion, confirmation of these preliminary translational analyses may be possible in future pooled analyses. This will include a deeper evaluation of the potentially complex inter-relationship between immunological components of the tumor microenvironment and dynamic temporal changes in afami-cel phenotype and function after infusion.

In summary, our findings demonstrate that afami-cel was well tolerated and suggest that it could be a promising therapy for patients with metastatic SS who have received prior ifosfamide treatment. Limitations to the interpretation of the response rate in SS include the small sample size and confounding effect of LD. Nevertheless, the encouraging results warrant further study, and additional data in metastatic SS will be evaluated in the phase 2 SPEARHEAD-1 trial^16^. Investigation into next-generation T cell therapies with enhanced cytolytic and immunological properties for a range of MAGE-A4^+^ epithelial solid tumors has commenced (NCT04044859 and NCT04752358).

## Methods

### Study design and participants

This open-label, phase 1 trial was conducted at multiple centers in North America to evaluate the safety of afami-cel in HLA-A*02^+^ patients with MAGE-A4-expressing solid cancers.

All patients underwent two pre-screening evaluations using different screening protocols (NCT02636855): (1) HLA testing for at least one HLA-A2 inclusion allele (HLA-A*02:01, HLA-A*02:02, HLA-A*02:03, HLA-A*02:06 or HLA-A*02:09) and absence of the exclusion allele HLA-A*02:05; and (2) MAGE-A4 tumor biopsy testing using an anti-MAGE-A4 immunohistochemistry (IHC) assay (Supplementary Table 1). Only patients with an appropriate HLA-A2 genotype and whose tumor expressed the MAGE-A4 antigen above the specified cutoff level were eligible to undergo screening for this study.

### Inclusion criteria

Patients must have voluntarily agreed to participate by giving written informed consent in accordance with International Council on Harmonization (ICH) Good Clinical Practice (GCP) guidelines and applicable local regulations. Patients must have agreed to abide by all protocol-required procedures, including study-related assessments and management by the treating institution for the duration of the study and LTFU phase. Patients were ≥18 years to ≤75 years of age at the time of signing the study informed consent.

Patients must have had histologically confirmed diagnosis of any one of the following cancers: (1) urothelial cancer (transitional cell cancer of the bladder, ureter, urethra or renal pelvis); (2) melanoma; (3) squamous cell carcinoma of the head and neck; (4) ovarian cancer; (5) NSCLC (squamous, adenosquamous, adenocarcinoma or large cell); (6) esophageal cancer (squamous and adenocarcinoma); (7) gastric cancer; (8) SS; or (9) MRCLS. Patients must have had measurable disease according to RECIST version 1.1 criteria before LD. Measurable disease was not required before leukapheresis. Patients must have had the following disease-specific requirements for their tumor type (note: there was no limit to the number of therapies before study entry). (1) Inoperable or metastatic (advanced) urothelial cancer: patients must have received at least one prior systemic therapy in the adjuvant or metastatic setting; may have received treatment with a programmed cell death protein 1 (PD-1) or programmed death-ligand 1 (PD-L1) inhibitor. (2) Inoperable or metastatic (advanced) melanoma: patients must have received, were intolerant to or refused a cytotoxic T lymphocyte-associated protein 4 inhibitor (ipilimumab) or a PD-1 inhibitor (nivolumab or pembrolizumab) as monotherapy or a combination of ipilumumab and nivolumab. Patients must have received or were intolerant to a B-Raf proto-oncogene (BRAF) inhibitor or the combination of BRAF and mitogen-activated protein kinase inhibitors for BRAF(V600) mutant melanoma. (3) Inoperable or metastatic (advanced) squamous cell head and neck cancer: patients must have received a platinum-containing chemotherapy for treatment of primary tumor in adjuvant, locally advanced or metastatic settings, were intolerant to or refused such treatment. They may have received prior immunotherapy. (4) Inoperable or metastatic (advanced) ovarian, primary peritoneal or fallopian tube carcinoma: patients must have received platinum-containing chemotherapy and had platinum-refractory or platinum-resistant disease. If platinum-sensitive disease, patients should have received at least two lines of chemotherapy. Patients may have received poly (adenosine diphosphate-ribose) polymerase inhibitors, bevacizumab or immunotherapy. (5) Histologically or cytologically confirmed diagnosis of advanced NSCLC (Stage IIIB or Stage IV) or recurrent disease (squamous, adenosquamous, adenocarcinoma or large cell carcinoma): patients whose tumors were known to have epidermal growth factor receptor (EGFR) mutations or anaplastic lymphoma kinase (ALK) gene rearrangements must have failed (PD or unacceptable toxicity) prior EGFR inhibitor or ALK tyrosine kinase inhibitor, respectively. Patients with ROS-1^+^ tumors must have failed an ALK inhibitor (crizotinib). Patients had received or are receiving at least one line of prior therapy. Patients may have received PD-1 inhibitors. There was no limit on lines of prior anti-cancer therapies. (6) Inoperable or metastatic (advanced) squamous or adenocarcinoma of the esophagus, gastroesophageal junction or gastric cancer: patients must have received, were intolerant to or refused at least one fluorouracil (5-FU) and/or platinum-containing regimen. Patients whose tumors were known to have HER2/neu amplification must have failed (PD or unacceptable toxicity) or refused trastuzumab. Patients may have received ramucirumab. (7) Advanced (metastatic or inoperable) SS or high-grade MRCLS confirmed by histology or cytogenetics: patients with SS must have previously received an anthracycline-containing regimen. Patients who were intolerant to anthracycline may have received ifosfamide alone. Patients with MRCLS must have previously received or be intolerant to an anthracycline-containing regimen.

Patients were HLA-A*02^+^ (this determination was made under screening protocol ADP-0000-001). The sponsor reviewed the results of HLA typing for inclusion and exclusion alleles and adjudicated patient eligibility based on HLA results. Patients’ tumors (either an archival specimen or a fresh biopsy) showed expression of the MAGE-A4 RNA or protein. All samples must have been reviewed by an Adaptimmune-designated central laboratory confirming expression. Patients had anticipated life expectancy of >6 months before leukapheresis and >3 months before LD. Patients had an Eastern Cooperative Oncology Group (ECOG) performance status of 0–1. Patients had a left ventricular ejection fraction ≥50%. Patients were fit for leukapheresis and had adequate venous access for the cell collection. Female patients of childbearing potential (FCBPs) must have had a negative urine or serum pregnancy test (note: FCBP was defined as pre-menopausal and not surgically sterilized). FCBPs must have agreed to use maximally effective birth control or to abstain from heterosexual activity throughout the study, starting at the first dose of chemotherapy, for 12 months after receiving the investigational product or 4 months after there was no evidence of persistence/gene-modified cells in the patient’s blood, whichever was longer. Male patients must have been surgically sterile or agreed to use a double-barrier contraception method or abstain from heterosexual activity with an FCBP starting at the first dose of chemotherapy and for 4 months thereafter. Patients must have had adequate organ function as indicated by laboratory values (Supplementary Table 11).

### Exclusion criteria

Patients who were HLA-A*02:05^+^ in either allele, those with HLA-A*02:07 as the sole HLA-A*02 allele and those with any A*02 null allele (designated with an ‘N’; for example, A*02:32N) as the sole HLA-A*02 allele were excluded from participating in the study. Patients must not have received or planned to receive excluded therapy/treatment before leukapheresis or LD chemotherapy (Supplementary Table 12). Patients with toxicity from previous anti-cancer therapy must have recovered to Grade ≤1 before enrollment (except for non-clinically significant toxicities—for example, alopecia and vitiligo). Patients with Grade 2 toxicities that were deemed stable or irreversible (for example, peripheral neuropathy) could have been enrolled.

Patients must not have had a history of allergic reactions attributed to compounds of similar chemical or biologic composition to fludarabine, cyclophosphamide or other agents used in the study. Patients must not have had a history of chronic or recurrent (within the last year before screening) severe autoimmune or immune-mediated disease requiring steroids or other immunosuppressive treatments, including anti-tumor necrosis factor (TNF) agents. Patients must not have had major surgery within 4 weeks before LD; patients should have been fully recovered from any surgical-related toxicities.

Patients with a prior history of symptomatic central nervous system (CNS) metastases must have received treatment (that is, stereotactic radiosurgery (SRS), whole-brain radiation therapy (WBRT) or surgery) and been neurologically stable for ≥1 month, not requiring anti-seizure medications and off steroids for ≥14 days before leukapheresis and LD. Patients with asymptomatic CNS metastatic disease without associated edema, shift, requirement for steroids or anti-seizure medications were eligible. If such a patient received SRS or WBRT, a minimum period of 2 weeks would be needed to lapse between the therapy and LD. Patients with leptomeningeal disease or carcinomatous meningitis were not eligible. Patients must not have had any other active malignancy besides the tumor under study within 3 years before screening. Exceptions were adequately treated malignancies not likely to require therapy (for example, completely resected non-melanomatous skin carcinoma or successfully treated in situ carcinoma). Newly identified prostate cancers found during cytoprostatectomy were permitted. Patients must have been in complete remission from prior malignancy to be eligible to enter the study.

Patients must not have had an electrocardiogram (ECG) showing clinically significant abnormality at screening or showing an average corrected QT interval ≥450 ms in males and ≥470 ms in females (≥480 ms for patients with bundle branch block) over three consecutive ECGs. Either Fridericia’s or Bazett’s formula may have been used to correct the QT interval. Patients must not have had uncontrolled intercurrent illness, including, but not limited to, ongoing or active infection; clinically significant cardiac disease defined by New York Heart Association Class of Heart Failure >1; uncontrolled clinically significant arrhythmia in the last 6 months or acute coronary syndrome (angina or myocardial infarction) in the last 6 months; interstitial lung disease (pneumonitis); history of pneumonectomy or chronic obstructive pulmonary disease with at least one exacerbation within 1 year before the screening visit that required treatment with systemic corticosteroids or resulted in hospitalization; pre-existing active skin disorders of Grade ≥2 severity; current uncontrolled hypertension despite optimal medical therapy; or a history of stroke or CNS bleeding, transient ischemic attack or reversible ischemic neurologic deficit within the last 6 months. Patients who, in the opinion of the investigator, were unlikely to fully comply with protocol requirements were ineligible. Patients must not have been pregnant or breastfeeding.

Patients must not have had active infection with human immunodeficiency virus (HIV), hepatitis B virus, hepatitis C virus (HCV) or human T-lymphotropic virus (HTLV). Patients with positive serology for HIV were excluded. Patients who were negative for hepatitis B surface antigen but positive for hepatitis B core antibody must have had undetectable hepatitis B DNA and received prophylaxis against viral reactivation. Prophylaxis should have been initiated before LD therapy and continued for 6 months. Patients who were positive for HCV antibody were screened for HCV RNA by any reverse transcription polymerase chain reaction (PCR) or branched DNA assay. If HCV antibody was positive, eligibility was determined based on a negative screening RNA value. Patients with positive serology for HTLV 1 or 2 were excluded. Re-screening for infectious disease markers was not required at baseline (before LD).

### Procedures

#### Study conduct

This trial was conducted in accordance with the Declaration of Helsinki and ICH GCP guidelines. The protocol was approved by local or independent institutional review boards at each trial center (Supplementary Table 13). Written informed consent was obtained from each patient. No compensation was provided for study participation. Participants may have received reimbursement for any costs incurred as a result of study participation. A Safety Review Committee assessed safety signals during dose escalation and adjudicated DLTs. The severity of TEAEs was graded according to National Cancer Institute Common Terminology Criteria for Adverse Events version 5.0. CRS was defined and graded according to criteria developed by Lee et al.^17^. Neurologic toxic effects were graded according to Chimeric Antigen Receptor T-cell Therapy-Associated Toxicity 10-point neurological assessment (CARTOX-10) criteria for cell-associated encephalopathy^18^.

#### Screening study (NCT02636855)

A screening study was initiated to pre-screen patients ≥18 years to ≤75 years of age with advanced solid tumors for the presence of inclusion and exclusion alleles (NCT02636855) for referral to this phase 1 trial (NCT03132922). HLA typing was performed using the 510(k)-cleared SeCore high-resolution sequence-based typing system for Class I loci (A, B and C) and uTYPE software (One Lambda Inc., Thermo Fisher Scientific). Central laboratory testing was performed using blood samples at the Histocompatibility/Molecular Genetics Laboratory at the American Red Cross in Philadelphia, Pennsylvania, which is accredited by the American Society for Histocompatibility and Immunogenetics.

For MAGE-A4 antigen expression, centralized testing by IHC was performed at HistoGeneX using an analytically validated and clinical laboratory improvement amendments (CLIA)-certified clinical trial assay. Positivity was determined by a pathologist based on the percentage of positive cells and intensity of expression, as determined by a percent score (P-score) (range, 0–100%) for IHC staining. P-score was defined as stained cell percentage at 1+ intensity + stained cell percentage at 2+ intensity + stained cell percentage at 3+ intensity. During the conduct of patient pre-screening in the expansion group, the study protocol was amended for SS and MRCLS to use a higher cutoff of ≥30% of cells that were of 2+ and/or 3+ intensity; a subsequent amendment was made to use this higher cutoff for all other tumor types in the expansion group. For translational data analyses, MAGE-A4 expression was presented as an H-score, which was defined as: (% stained cells at 0) × 0 + (% stained cells at 1+) × 1 + (% stained cells at 2+) × 2 + (% stained cells at 3+) × 3. The H-score range is 0–300 (refs. 19–21).

#### Phase 1 study (NCT03132922)

Patients who met the pre-screening requirements were then screened for eligibility criteria for this phase 1 trial (NCT03132922), including age ≥18 years to ≤75 years, ECOG score of 0 or 1, measurable disease per RECIST version 1.1 and adequate organ function, including creatinine clearance (CrCl) ≥60 ml min^−1^. Eligible patients entered the interventional phase and then the LTFU phase (Extended Data Fig. 1). Patients who ended the interventional phase continued in the LTFU phase for long-term monitoring for potential gene-therapy-related delayed AEs for 15 years after infusion.

Leukapheresis was performed to obtain CD3^+^ T cells for afami-cel manufacture. Systemic bridging therapy was permissible before LD chemotherapy.

Patients received LD chemotherapy consisting of fludarabine 30 mg/m^2^ and cyclophosphamide 600 mg/m^2^ on days −7, −6 and −5 before afami-cel infusion (Supplementary Table 14). Fludarabine doses were adjusted per baseline CrCl: 30 mg/m^2^ for patients with CrCl ≥80 ml min^−1^ and 20 mg/m^2^ for patients with CrCl ≥60 ml min^−1^ and <80 ml min^−1^; patients with CrCl <60 ml min^−1^ were ineligible for study treatment. Granulocyte colony-stimulating factor could be administered 24 hours after the last fludarabine infusion.

This phase 1 trial was conducted using a 3 + 3 design and involved dose escalation of afami-cel across dose Groups 1–3 and an expansion group. Dose ranges (total transduced cell number) were 0.08 × 10^9^ to 0.12 × 10^9^ cells for Group 1 (*n* = 3, all ovarian cancer); 0.5 × 10^9^ to 1.2 × 10^9^ cells for Group 2 (*n* = 3, all ovarian cancer); 1.2 × 10^9^ to 6.0 × 10^9^ cells for Group 3 (*n* = 3; one esophagogastric junction (EGJ) cancer, one ovarian cancer and one SS); and 1.2 × 10^9^ to 10 × 10^9^ cells for the expansion group (*n* = 29, one EGJ, one esophageal, three head and neck, one melanoma, five MRCLS, two ovarian, 15 SS and two urothelial). Group 1 received LD chemotherapy of cyclophosphamide (600 mg/m^2^/day) on days −7, −6 and −5 and fludarabine (30 mg/m^2^/day) on days −7, −6 and −5. Group 2 received LD chemotherapy of cyclophosphamide (600 mg/m^2^/day) on days −7, −6 and −5 and fludarabine (30 mg/m^2^/day) on days −7, −6 and −5. Group 3 received LD chemotherapy of cyclophosphamide (600 mg/m^2^/day) on days −7, −6 and −5 and fludarabine (30 mg/m^2^/day) on days −7, −6, −5 and −4. Most patients in the expansion group (*n* = 22) received LD chemotherapy of cyclophosphamide (600 mg/m^2^/day) on days −7, −6 and −5 and fludarabine (30 mg/m^2^/day) on days −7, −6, −5 and −4. Seven patients in the expansion group received the higher LD chemotherapy of cyclophosphamide (1,800 mg/m^2^/day) on days −3 and −2 and in combination with fludarabine (30 mg/m^2^/day) on days −5, −4, −3 and −2. DLTs were evaluated before each dose escalation, with doses progressively increased to 1.2 × 10^9^ to 10.0 × 10^9^ cells in the expansion group. Eligible patients could receive a second cell infusion after disease progression after a confirmed response.

Evaluation of safety and tolerability was conducted at each study visit as follows: baseline; days −7 to −3; days 1–5; days 8, 15, 22, 29, 36, 43, 57, 71, 85, 127 and 169; every 3 months until year 2; every 6 months from years 2 to 5 or until disease progression; and at completion.

#### Engineering of afami-cel

Afami-cel was engineered using the Adaptimmune p1.5, p1.5.1 and p1.6.1 manufacturing processes. The p1.5 process used the COBE 2991 Cell Processor cell washer (Terumo BCT) and Xuri Cell Expansion System W25 bioreactor (Cytivia). During the trial, process improvements were made to the volume of material processed, with the process being updated to p1.5.1. Further improvements were made during the trial, with the COBE 2991 Cell Processor being replaced by the Sepax C-Pro Cell Processing System (Cytivia) for the p1.6.1 manufacturing process. Along with changes to the cell manufacturing process over the course of the trial, there were also multiple sources of lentiviral vector. The initial source of vector was manufactured by City of Hope and used an adherent-based manufacturing process. The lentiviral vector sourced later in the trial was manufactured by Lentigen and used a suspension-based manufacturing process. The median vector copy number in the product was 4.5 per transduced cell (range, 2.2–8.5 per transduced cell); the median transduction efficiency for the product was 53.3% (range, 25.5–80%); and the median number of cells produced during the manufacturing process was 20.7 × 10^9^ total nucleated cells (range, 5.0 × 10^9^ to 42.2 × 10^9^). The final cell product release was contingent on several different specifications, including CD3^+^ ≥80% of cells, vector copy number <12.0 and cell viability ≥70%; the final afami-cel product was cryopreserved in 5% dimethyl sulfoxide and thawed before intravenous administration.

#### Immunophenotyping and cytotoxicity

Immunophenotypic profiling of MP and PBMC samples was performed by flow cytometry, using multicolor staining panels (CD3 (SK7), CD4 (SK3) and CD8 (SK1), BD Biosciences; CD45RA (HI100) and CCR7 (G043H7), BioLegend; and Live/Dead Fix Aqua, Thermo Fisher Scientific). For all samples, in the CD3^+^ live population, subsets of CD4^+^ and CD8^+^ cells (assessed for transduction using a major histocompatibility complex dextramer reagent) were further classified into memory subtypes by expression of CCR7 and CD45RA: central (CM, CD45RA^−^CCR7^+^), effector (EM, CD45RA^−^CCR7^−^), terminally differentiated (EMRA, CD45RA^+^CCR7^−^) and stem cell like (SCM, CD45RA^+^CCR7^+^) (Supplementary Fig. 1). Two-sided paired Wilcoxon test *P* values are shown, linking compared cell types. Immunophenotyping data were collected using BD FACSDiva 9.0 software and analyzed in FlowJo 9.9 (BD). Antibody dilutions are reported in Supplementary Table 15.

Functionality of T cells in samples of MP administered were profiled in vitro using an exploratory cellular cytotoxicity assay. Tumor cell growth (A375nucGFP^+^) was tracked using Incucyte image acquisition and analysis (Sartorius). The same control MP sample was profiled in parallel with each sample as a continuity control. Tumor cell growth data were calculated at 72 hours and 125 hours for MP, and degree of tumor cell killing at each timepoint was represented as percentage killing relative to no T cell controls. Cytotoxicity assay data were performed via analysis of collected images within Sartorius Incucyte Zoom software 2019B Rev2.

#### Cell persistence

Persistence of transduced T cells in PBMCs after infusion was measured using quantitative PCR (qPCR) specific for the packaging signal sequence present in the vector genome. This sequence, absent from the human genome, is integrated in the genome of the transduced host T cell and, thus, acts as a genetic marker specific for transduced cells. Persistence is expressed as the number of vector copies per microgram of genomic DNA from PBMCs.

#### Lentiviral vector integration site analysis

Lentiviral vector integration sites were identified in DNA extracted from the MPs (transduced T cells) and from PBMC samples collected between 6 months and 15 months after infusion in patients exhibiting high levels of long-term persistence (>1% of transduced PBMCs based on Psi qPCR). The identification and quantification of each integration site was performed using next-generation sequencing using the SonicAbundance method^22^.

#### Soluble biomarkers

To evaluate peripheral immune marker profiles, patient-derived serum samples taken before and after afami-cel infusion for the mITT population (*N* = 38) were analyzed. Using multiplex assays on the Meso Scale Discovery platform, levels (pg ml^−1^) of a panel of 22 markers were determined: IFNγ, GMCSF, TNF-α, TNF-β, vascular endothelial growth factor, IL-1α, IL-1β, IL1-RA, IL-2, IL-2Rα, IL-4, IL-5, IL-6, IL-7, IL-8, IL-10, IL-12p70, IL-13, IL-15, IL-16, IL-17A and IL-12/IL-23p40. Further exploratory analyses measured 92 immuno-oncology-related human proteins simultaneously using Olink IMMUNO-ONCOLOGY, based on Proximity Extension Assay technology (Olink Bioscience) Detection was performed using the Fluidigm Biomark or the Fluidigm Biomark HD system. Data are reported as Normalized Protein eXpression unit. Data figures were generated using RStudio (and R version 4.2.1) and appropriate R script packages.

#### Afami-cel RNA in situ hybridization and CD3 IHC

RNA in situ hybridization for MAGE-A4 coding SPEAR (Advanced Cell Diagnostics) was performed on the Ventana Discovery Ultra automation platform (Roche Diagnostics) using the RNAscope 2.5 LS Red kit (Advanced Cell Diagnostics, 322150) according to the manufacturer’s instructions. In brief, 4-μm formalin-fixed, paraffin-embedded (FFPE) tissue sections were pre-treated with heat and protease before hybridization with the target oligo probes. Pre-amplifier, amplifier and alkaline phosphatase-labeled oligos were then hybridized sequentially. RNAscope assay was followed by CD3 chromogenic precipitate IHC (anti-CD3 (2GV6) rabbit monoclonal primary antibody, 790-4341, CONFIRM, Roche Diagnostics) using the DISCOVERY TEAL HRP detection kit (Roche Diagnostics, 08254338001). Each sample was quality controlled for RNA integrity with an RNAscope probe specific to peptidylpropyl isomerase B (PPIB) RNA (Hs-PPIB, 313909). Specific RNA staining signal was identified as red punctate dots, and CD3 was identified by the teal signal. Samples were counterstained with hematoxylin.

The whole slides were scanned using the AxioScan.Z1 microscope slide scanner and analyzed using HALO image analysis software (Indica Labs). Images were annotated and analyzed using Indica Labs-ISH version 3.3.9 algorithm, with CD3 staining as the nuclear stain, resulting in CD3^+^ cell count and MAGE-A4 SPEAR percentage positivity.

#### MAGE-A4 IHC

MAGE-A4 (OriGene, TA505362) IHC staining was performed on 4-μm sections of FFPE biopsy tissues. Staining was performed and scored at a laboratory certified by the CLIA and accredited by the Belgian Accreditation Organization and College of American Pathologists (HistoGeneX).

#### Multiplex immunofluorescence staining

Multiplex immunofluorescence staining was performed using Ultivue FixVUE 8-plex multiplex immunostaining kit (CD3, CD4, CD8, FoxP3, granzyme B, Ki67, PanCK/Sox10 and PD-L1) and conducted on a Leica Bond Rx (Leica Biosystems) fully automated immunostainer, according to the kit protocol. The whole slides were scanned using an AxioScan.Z1 microscope slide scanner; images were stacked using Ultivue’s Ultistacker software for InSituPlex image co-registration (version 1.0); and stacked image datasets were analyzed using HALO image analysis software. Images were annotated and analyzed using High Plex FL version 3.2.1 algorithm (Indica Labs), resulting in co-localization output for key immunophenotypes, such as CD4 T cells (CD3^+^CD4^+^), cytotoxic T cells (CD3^+^CD8^+^), regulatory T cells (CD3^+^CD4^+^FoxP3^+^) and malignant cells (PanCK/Sox10^+^) as well as activated (granzyme B^+^), proliferating (Ki67^+^) and PD-L1^+^ combinations of these phenotypes. Spatial analysis plots were generated, using HALO Spatial Analysis module, by selected combinations of these immunophenotypes. The generated spatial plots represent spatial organization and distribution of malignant cells to cytotoxic and regulatory T cells in a selected region of interest. Semi-quantitative analysis of cell numbers (per square millimeter of tumor tissue area), for each of immunophenotype, was performed by exporting the summary results from HALO and importing these data into RStudio (and R version 4.2.1) to generate the relevant heat maps and line plots.

#### NanoString

FFPE-derived RNA samples were analyzed on the NanoString platform using nCounter PanCancer Immune Profiling Panel (XT-CSO-HIP1-12; NS_CancerImmune_V1.1). Sample quality control was carried out as per the manufacturer’s instructions. Background levels were determined for each sample using mean plus two standard deviations of the included negative control counts. Background subtracted counts were normalized using geometric means of 27 housekeeping genes. log_2_-normalized counts for the ‘T cell exhaustion’ gene list was compiled based on published literature^23^. ‘Negative regulation of T cell-mediated immunity’ gene list was obtained from the corresponding Gene Ontology term^24,25^. Normalized counts were analyzed using the gene set variation analysis algorithm described previously^26^. Gene lists were extracted from NanoString panel annotation for cell types and immune response categories, excluding any gene list with fewer than three associated genes. Data figures were generated using the ComplexHeatmap R package^27^. The NanoString data are available publicly at https://www.ncbi.nlm.nih.gov/geo/query/acc.cgi?acc=GSE202156.

#### Outcomes

The primary objective was evaluation of safety and tolerability with endpoints including TEAEs, serious AEs, DLTs and detection of replication-competent lentivirus (RCL). Secondary endpoints included ORR confirmed by RECIST version 1.1, BOR, TTR, DoR, duration of SD, PFS and OS. Exploratory objectives included evaluation of cell persistence and cytokines. Exploratory endpoints included correlation of persistence, phenotype and functionality of transduced (afami-cel) and non-transduced T cells in the peripheral blood and/or tumor in response to treatment and safety; determination of target antigen expression, genes related to antigen processing/presentation and cell surface co-stimulatory ligands; and evaluation of serum cytokines (that is, IL-6). The following exploratory endpoints were not analyzed: evaluation of anti-tumor antibodies or candidate biomarkers from plasma-derived exosomes and cell-free DNA. Owing to the preliminary nature of the data collected before the data cutoff, limited correlative analyses were reported in relation to response to treatment and safety. Two patients received a second infusion; they were non-responders. Therefore, ORR was not evaluated for the second infusion.

### Statistical analysis

The sample size for this study was not pre-specified. This phase 1 study was not statistically powered to evaluate either safety or efficacy; hence, the data were summarized descriptively. No formal hypothesis testing was planned. ORR was defined as the proportion of patients with a BOR of confirmed complete response or partial response per RECIST version 1.1 relative to the total number of patients in the corresponding analysis population. DCR was defined as the percentage of patients whose disease reduced or remained stable as per RECIST version 1.1. PFS, OS, TTR and DoR with 95% CIs were estimated using Kaplan–Meier methods. BOR per RECIST version 1.1 and ORR with 95% CIs were summarized. Censoring of data for DoR and PFS was based on US Food and Drug Administration censoring rules (https://www.fda.gov/media/71195/download)^28,29^. Censoring will occur as follows for DoR:

- If there are no adequate tumor assessments after the patient achieved confirmed response, the DoR will be censored and have a duration set to 1.
- If a patient is known to be alive and progression free, DoR will be censored on the day of the last adequate tumor assessment.
- Use of active curative anti-cancer therapy after T cell infusion (that is, before disease progression) will also be considered as meeting the PD criterion. If a patient is given subsequent curative anti-tumor therapies, curative anti-cancer therapies and curative anti-cancer surgeries other than the study treatment (not including approved palliative radiation and diagnostic procedures, such as surgical biopsies before PD or death), DoR will be censored on the date of the last progression-free tumor assessment before the start date of the anti-tumor treatment.
- If a patient discontinues the interventional phase of the study before PD, DoR will be censored on the date of last adequate progression-free tumor assessment.
- If a patient misses two or more consecutive post-baseline tumor assessments and the following assessment is a PD, or if a patient misses two or more consecutive post-baseline tumor assessments and then dies, DoR will be censored on the date of the last adequate tumor assessment.

Censoring will occur as follows for PFS:

- If a patient has an inadequate baseline scan, PFS will be censored and have a duration set to 1.
- If there are no adequate post-baseline tumor assessments after T cell infusion or date of death recorded, PFS will be censored and have a duration set to 1.
- If a patient is known to be alive and progression free, PFS will be censored on the day of the last adequate tumor assessment.
- To note, use of active curative anti-cancer therapy after T cell infusion (that is, before disease progression) will also be considered as meeting the PD criterion. If a patient is given subsequent curative anti-tumor therapies, curative anti-cancer therapies and curative anti-cancer surgeries other than the study treatment (not including approved palliative radiation and diagnostic procedures, such as surgical biopsies before PD or death), DoR will be censored on the date of the last progression-free tumor assessment before the start date of the anti-tumor treatment.
- If a patient discontinues the interventional phase before PD, PFS will be censored on the date of the last adequate tumor assessment.
- If a patient misses two or more consecutive post-baseline tumor assessments and the following assessment is a PD, or if a patient misses two or more consecutive post-baseline tumor assessments and then dies, PFS will be censored on the date of the last adequate tumor assessment.

Safety data were summarized using descriptive statistics. Persistence of afami-cel, RCL and cytokines were summarized descriptively. Analyses were performed with SAS software version 9.4.

### Reporting summary

Further information on research design is available in the Nature Portfolio Reporting Summary linked to this article.

## Online content

Any methods, additional references, Nature Portfolio reporting summaries, source data, extended data, supplementary information, acknowledgements, peer review information; details of author contributions and competing interests; and statements of data and code availability are available at 10.1038/s41591-022-02128-z.

## Supplementary information

Supplementary Information List of investigators and study sites, Supplementary Fig. 1 and Supplementary Tables 1–15

Reporting Summary

## Data Availability

The NanoString data are available publicly at https://www.ncbi.nlm.nih.gov/geo/query/acc.cgi?acc=GSE202156. The clinical datasets generated and/or analyzed during the current study are available upon reasonable request from the corresponding author for research only, non-commercial purposes. Such datasets include study protocol, statistical analysis plan, individual participant data that underlie the results reported in this article after de-identification (text, tables, figures and appendices) as well as supporting documentation as required. Restrictions relating to patient confidentiality and consent will be maintained by aggregating and anonymizing identifiable patient data. The clinical data will be available beginning immediately after article publication and thereafter with no time limit. Requests should be sent in writing describing the nature of the proposed research and extent of data requirements. Data recipients are required to enter a formal data-sharing agreement that describes the conditions for release and requirements for data transfer, storage, archiving, publication and intellectual property. Requests should be directed to Dennis Williams and will be reviewed by the corresponding senior authors, D.S.H. and M.O.B., and by Adaptimmune. Responses will typically be provided within 60 days of the initial request.
