# Supplementary material for: Autologous T cell therapy for MAGE-A4+ solid cancers in HLA-A*02+ patients: a phase 1 trial
Source: Nat Med. 2023 Jan 9;29(1):104–14. doi: 10.1038/s41591-022-02128-z (PMC9873554; doi:10.1038/s41591-022-02128-z)
Supplement: Supplementary file 1 — List of investigators and study sites, Supplementary Fig. 1 and Supplementary Tables 1–15 [file 41591_2022_2128_MOESM1_ESM.pdf]

nature medicine

Article

https://doi.org/10.1038/s41591-022-02128-z

Autologous T cell therapy for MAGE-A4^{+} solid cancers in HLA-A*02^{+} patients: a phase 1 trial

In the format provided by the authors and unedited

Table of Contents

List of Investigators and Study Sites 2
Supplementary Figures....3
Supplementary Figure 1. Example of flow cytometry gating strategy....3
Supplementary Tables 4
Supplementary Table 1. Summary of HLA status and antigen MAGE-A4 expression at screening (modified intent-to-treat population; N=38) 4
Supplementary Table 2. Summary of patient characteristics (modified intent-to-treat population; N=38) 5
Supplementary Table 3. Summary of synovial sarcoma subgroup characteristics....7
Supplementary Table 4. Incidence of cytokine release syndrome (modified intent-to-treat population; N=38)....8
Supplementary Table 5. Patients with SS administered two afami-cel infusions....9
Supplementary Table 6. Summary of overall response rate and best overall response (modified intent-to-treat population; N=38)....10
Supplementary Table 7. Integration site analysis to evaluate clonality status....11
Supplementary Table 8. Serum proteins peak and AUC concentrations for patients with cytokine release syndrome versus patients without cytokine release syndrome....12
Supplementary Table 9. Correlation of peak serum level with best percentage change in target lesion SLD 13
Supplementary Table 10. Afami-cel dose, vector copy number, tumor MAGE-A4 expression and serum IFNγ profiles in ovarian cancer across all dose groups 14
Supplementary Table 11. Laboratory values to define adequate organ function 15
Supplementary Table 12. Prohibited therapy/treatment prior to leukapheresis or lymphodepleting chemotherapy 16
Supplementary Table 13. List of institutional review boards and ethics committees....18
Supplementary Table 14. Fludarabine and cyclophosphamide treatment schema: all dose groups 20
Supplementary Table 15. Antibody dilutions....21

List of Investigators and Study Sites

Investigator	Location	Number of Patients Treated
David S Hong, MD	The University of Texas MD Anderson Cancer Center, Houston, TX, USA	16
Marcus Butler, MD	Princess Margaret Cancer Centre, Toronto, ON, Canada	10
Brian Van Tine, MD	Washington University School of Medicine, St. Louis, MO, USA	7
Anthony Olszanski, MD	Fox Chase Cancer Center, Philadelphia, PA, USA	2
David A Liebner, MD	James Cancer Hospital and Solove Research Institute, Columbus, OH, USA	1
Melissa L Johnson, MD	The Sarah Cannon Research Institute, Nashville, TN, USA	1
Adekunle Odunsi, MD, PhD	Roswell Park Cancer Institute, Buffalo, NY, USA	1
Jeffrey Clarke, MD	Duke University Medical Cancer Center, Durham, NC, USA	0
Mihaela Druta, MD	H. Lee Moffitt Cancer and Research Institute, Tampa, FL, USA	0
Brian M Slomovitz, MD	Sylvester Comprehensive Cancer Center, Miami, FL, USA	0

Supplementary Figures

Supplementary Figure 1. Example of flow cytometry gating strategy

Gating was applied according to the following hierarchy. Live cells excluding Aqua dead cell stain were gated. Within these a lymphocyte gate was applied as shown. Single lymphocytes were gated from here and CD3+ cells gated therein. From the CD3+ population CD4+ and CD8+ gates were applied and within these gated for TCR status via a Dextramer reagent. From these transduced and non-transduced CD4 and CD8 gates, CD45RA and CCR7 quadrants were applied to select memory subsets

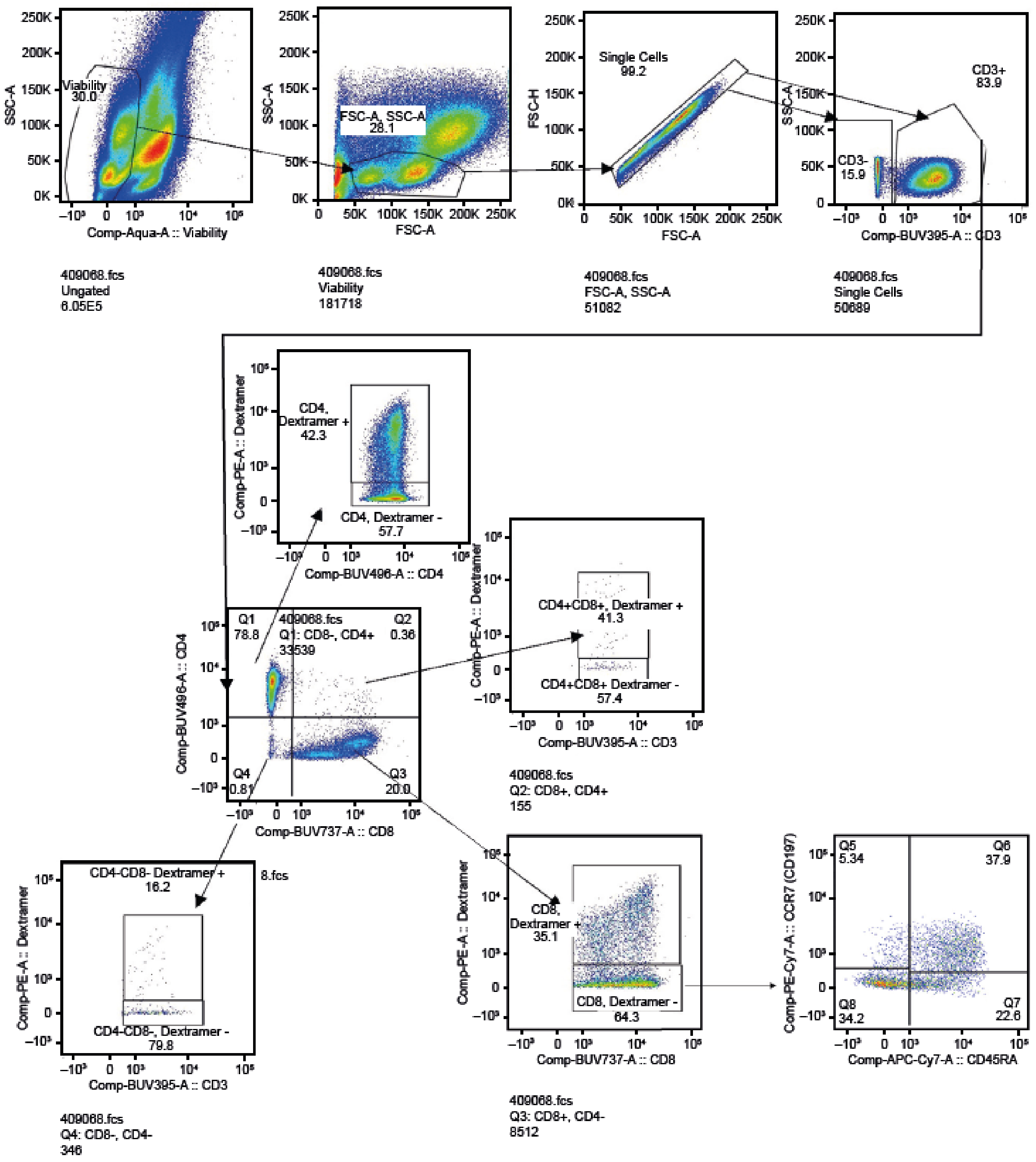

Supplementary Tables

Supplementary Table 1. Summary of HLA status and antigen MAGE-A4 expression at screening (modified intent-to-treat population; N=38)

Parameter	Category	Statistic	Overall (N=38)
HLA inclusion allele[a]	HLA-A*02:01	n (%)	35 (92.1) [a]
	HLA-A*02:03	n (%)	1 (2.6)
	HLA-A*02:06	n (%)	2 (5.3)
MAGE-A4 expression H-score[b]	NA	Mean (standard deviation)	176.8 (98.25)
		Median	189.0
		Min, max	15, 300

[a] 29 patients had A*02:01 as allele 1 and 12 patients had A*02:01 as allele 2 (the allele with the lower number is allele 1 by default). Of these, 6 patients had A*02:01 as both allele 1 and allele 2 (homozygous) and were counted once

[b] H-score = (% stained cells at 0) × 0 + (% stained cells at 1+) × 1 + (% stained cells at 2+) × 2 + (% stained cells at 3+) × 3; The H score range is 0-300

HLA, human leukocyte antigen; MAGE-A4, melanoma-associated antigen A4; NA, not applicable

Supplementary Table 2. Summary of patient characteristics (modified intent-to-treat population; N=38)

Parameter	Category	Statistic	Overall (N=38)
Age (years)		N	38
		Mean (standard deviation)	56.4 (12.59)
		Median	58.0
		Min, max	31, 78
Age categorization	<65 years	n (%)	28 (73.7)
	≥65 years	n (%)	10 (26.3)
Sex	Male	n (%)	22 (57.9)
	Female	n (%)	16 (42.1)
Height (cm)		N	33
		Mean (standard deviation)	172.0 (10.55)
		Median	173.0
		Min, max	147.0, 190.0
Weight (kg)		N	38
		Mean (standard deviation)	83.9 (22.55)
		Median	81.7
		Min, max	42.9, 148.8
BMI (kg/m^2)		N	33
		Mean (standard deviation)	27.4 (6.84)
		Median	26.0
		Min, max	15.5, 46.6
Ethnicity	Hispanic/Latino	n (%)	2 (5.3)
	Not Hispanic/Latino	n (%)	36 (94.7)
Race	White	n (%)	35 (92.1)
	Asian	n (%)	3 (7.9)
Geographical region	North America	n (%)	38 (100)
Primary tumor type	Esophageal	n (%)	2 (5.3)
	Gastric	n (%)	1 (2.6)
	Head and neck	n (%)	3 (7.9)
	Melanoma	n (%)	1 (2.6)
	Non-small-cell lung cancer	n (%)	2 (5.3)
	Ovarian	n (%)	9 (23.7)
	Urothelial	n (%)	2 (5.3)
	Myxoid/Round cell liposarcoma	n (%)	2 (5.3)
	Synovial sarcoma	n (%)	16 (42.1)
ECOG score	0	n (%)	13 (34.2)
	1	n (%)	25 (65.8)
Time from initial diagnosis to enrollment (months)[a]		Mean (standard deviation)	48.5 (40.23)
		Median	37.5
		Min, max	5.2, 203.2
		Mean (standard deviation)	51.4 (40.33)

Parameter	Category	Statistic	Overall (N=38)
Time from initial diagnosis to T-cell infusion (months) [b]		Median	40.4
		Min, max	7.3, 205.5
Prior lines of systemic therapy		Mean (standard deviation)	3.2 (1.88)
		Median	3.0
		Min, max	1, 8
Prior lines of systemic therapy (categorical)	1	n (%)	7 (18.4)
	2	n (%)	10 (26.3)
	3	n (%)	6 (15.8)
	≥4	n (%)	15 (39.5)
Prior anti-cancer therapy	Cytotoxic chemotherapy	n (%)	37 (97.4)
	Immunotherapy	n (%)	12 (31.6)
	Biological therapy	n (%)	1 (2.6)
	Small molecule/targeted therapy	n (%)	5 (13.2)
	Radiotherapy	n (%)	22 (57.9)
	Any surgery	n (%)	32 (84.2)
	Other therapy	n (%)	2 (5.3)
Bridging therapy	Cytotoxic chemotherapy	n (%)	21 (55.3)
	Immunotherapy	n (%)	2 (5.3)
	Small molecule/targeted therapy	n (%)	3 (7.9)
	Radiotherapy	n (%)	2 (5.3)
	Any surgery	n (%)	2 (5.3)
	Other therapy	n (%)	2 (5.3)
H-score[c]	NA	Mean (standard deviation)	176.8 (98.25)
		Median	189.0
		Min, max	15, 300
Total transduced cells ($\times 10^9$)		Mean (standard deviation)	6.1213 (3.422688)
		Median	6.4022
		Min, max	0.1, 9.9756

Note: Present height, weight, calculated BMI at baseline (not at screening)
[a] Time since initial Diagnosis in months is calculated as: (Date of signed ICF – date since initial diagnosis +1)*(12/365.25)
[b] Time from initial diagnosis to T-cell infusion in months is calculated as: (T-cell infusion date – date since initial diagnosis + 1)*(12/365.25)
[c] H-score = (% stained cells at 0) × 0 + (% stained cells at 1+) × 1 + (% stained cells at 2+) × 2 + (% stained cells at 3+) × 3; The H score range is 0-300
BMI, body mass index; ECOG, Eastern Cooperative Oncology Group; ICF, informed consent form; NA, not applicable

Supplementary Table 3. Summary of synovial sarcoma subgroup characteristics

Parameter	Category	Statistic	Synovial Sarcoma (N=16)
Age (years)		Median (range)	49.0 (31, 76)
Sex	Male	n (%)	10 (62.5)
	Female	n (%)	6 (37.5)
Race	White	n (%)	14 (87.5)
	Asian	n (%)	2 (12.5)
ECOG performance status	0	n (%)	10 (62.5)
	1	n (%)	6 (37.5)
Prior lines of systemic therapy		Median (range)	2.5 (1, 6)
H-score[a]		Median (range)	249 (60, 300)
Total transduced cells ($\times 10^9$)		Median (range)	9.28 (3.4, 10)

[a] H-score = (% stained cells at 0) × 0 + (% stained cells at 1+) × 1 + (% stained cells at 2+) × 2 + (% stained cells at 3+) × 3; The H score range is 0-300

ECOG, Eastern Cooperative Oncology Group

Supplementary Table 4. Incidence of cytokine release syndrome (modified intent-to-treat population; N=38)

<table>
<tr><th>Maximum grade</th><th>Onset day</th><th>Duration, days</th><th>Serious Adverse Event</th><th>Relationship to Treatment</th><th>Anti-IL-6/IL-6 Receptor</th></tr>
<tr><td>1</td><td>7</td><td>4</td><td>Yes</td><td>T-cell infusion</td><td>Tocilizumab</td></tr>
<tr><td rowspan="2">1[a]
2</td><td>4</td><td>2</td><td>No</td><td>T-cell infusion</td><td>No</td></tr>
<tr><td>6</td><td>7</td><td>No</td><td>T-cell infusion</td><td>Tocilizumab</td></tr>
<tr><td>2</td><td>9</td><td>7</td><td>Yes</td><td>T-cell infusion</td><td>No</td></tr>
<tr><td rowspan="2">2[b]
2</td><td>2</td><td>2</td><td>No</td><td>T-cell infusion</td><td>Tocilizumab</td></tr>
<tr><td>25</td><td>4</td><td>Yes</td><td>T-cell infusion</td><td>Tocilizumab</td></tr>
<tr><td>1</td><td>3</td><td>2</td><td>No</td><td>T-cell infusion</td><td>No</td></tr>
<tr><td>1</td><td>3</td><td>3</td><td>No</td><td>T-cell infusion and LD chemotherapy</td><td>No</td></tr>
<tr><td>1</td><td>2</td><td>5</td><td>No</td><td>T-cell infusion</td><td>Tocilizumab</td></tr>
<tr><td>2</td><td>9</td><td>6</td><td>Yes</td><td>T-cell infusion</td><td>No</td></tr>
<tr><td>1</td><td>3</td><td>1</td><td>No</td><td>T-cell infusion</td><td>No</td></tr>
<tr><td rowspan="2">1[a]
2</td><td>2</td><td>2</td><td>No</td><td>T-cell infusion</td><td>No</td></tr>
<tr><td>4</td><td>4</td><td>Yes</td><td>T-cell infusion</td><td>No</td></tr>
<tr><td>1</td><td>3</td><td>3</td><td>No</td><td>T-cell infusion</td><td>No</td></tr>
<tr><td rowspan="2">1[a]
1</td><td>1</td><td>1</td><td>No</td><td>T-cell infusion</td><td>No</td></tr>
<tr><td>3</td><td>11</td><td>No</td><td>T-cell infusion</td><td>No</td></tr>
<tr><td rowspan="3">1[a]
3
1</td><td>2</td><td>12</td><td>Yes</td><td>T-cell infusion</td><td>No</td></tr>
<tr><td>14</td><td>1</td><td>Yes</td><td>T-cell infusion</td><td>Tocilizumab</td></tr>
<tr><td>15</td><td>6</td><td>Yes</td><td>T-cell infusion</td><td>No</td></tr>
<tr><td>1</td><td>6</td><td>9</td><td>Yes</td><td>T-cell infusion and LD chemotherapy</td><td>Tocilizumab</td></tr>
<tr><td rowspan="2">3[a]
4</td><td>2</td><td>12</td><td>Yes</td><td>T-cell infusion</td><td rowspan="2">Tocilizumab and siltuximab</td></tr>
<tr><td>39</td><td>14</td><td>Yes</td><td>T-cell infusion</td></tr>
<tr><td>2</td><td>2</td><td>2</td><td>No</td><td>T-cell infusion</td><td>Tocilizumab</td></tr>
<tr><td rowspan="2">1[a]
2</td><td>3</td><td>1</td><td>No</td><td>T-cell infusion</td><td>No</td></tr>
<tr><td>4</td><td>3</td><td>No</td><td>T-cell infusion</td><td>No</td></tr>
<tr><td>1</td><td>4</td><td>2</td><td>No</td><td>T-cell infusion</td><td>Tocilizumab</td></tr>
<tr><td>2</td><td>2</td><td>7</td><td>Yes</td><td>T-cell infusion</td><td>Tocilizumab</td></tr>
<tr><td>1</td><td>2</td><td>2</td><td>No</td><td>T-cell infusion</td><td>Tocilizumab</td></tr>
<tr><td rowspan="2">1[a]
2</td><td>2</td><td>2</td><td>No</td><td>T-cell infusion</td><td>No</td></tr>
<tr><td>4</td><td>2</td><td>No</td><td>T-cell infusion</td><td>Tocilizumab</td></tr>
<tr><td colspan="6">[a] >1 separate CRS event reported in a single patient
[b] Patient treated with two separate afami-cel infusions
CRS, cytokine release syndrome; IL, interleukin; LD, lymphodepletion</td></tr>
</table>

Supplementary Table 5. Patients with SS administered two afami-cel infusions

		**1st Afami-cel Infusion**	**2nd Afami-cel Infusion***
Patient A	Date of Afami-cel Infusion	14 January 2019	12 August 2019
	MAGE-A4 H-Score	214 (screening)	37 (baseline)**
	Transduced Cell Dose	9.9×10^9	5.4×10^9
	Target Lesion Baseline SLD	20.4 cm<br>(large left lung pleural lesion crossing midline; see Figure 3 (upper panel))	21.4 cm
	CRS	Grade 2	Grade 2
	ICANS	No	No
	Prolonged Cytopenia at WK4	Hb and neutrophils	Hb and platelets
	SAEs	CRS Grade 2	Pulmonary embolism: Grade 3
	BOR	PR (-45% SLD reduction)	PD
	DoR	14.14 W	Not applicable
Patient B	Date of Afami-cel Infusion	28 January 2019	14 October 2019
	MAGE-A4 H-Score	286 (screening)	300 (baseline)
	Transduced Cell Dose	9.9×10^9	8.0×10^9
	Target Lesion Baseline SLD	24.0 cm	10.8 cm
	CRS	Grade 2	No
	ICANS	No	No
	Prolonged Cytopenia at WK4	No	No
	SAEs	Laryngeal hemorrhage: Grade 3	None
	BOR	PR (-86% SLD reduction)	SD
	DoR	12.28 W	Not applicable

*2nd afami-cel infusion was administered after confirmation of progressive disease from a 1st infusion that attained a PR; in both cases, 2nd T-cell infusions were manufactured from surplus cells collected at the initial leukapheresis and patients were administered Flu/Cy LD chemotherapy before 2nd T-cell infusion
**Decrease in MAGE-A4 expression at tumor progression is being evaluated as a mechanism of immune escape in the ongoing phase 2, SPEARHEAD-1, trial in SS and MRCLS
BOR, best overall response; CRS, cytokine release syndrome; Cy, cyclophosphamide; DoR duration of response; flu, Fludarabine; Hb, hemoglobin; ICANS, immune effector cell-associated neurotoxicity syndrome; MAGE-A4, melanoma-associated antigen A4; MRCLS, myxoid/round cell liposarcoma; PD, progressive disease; PR, partial response; PT, patient; SAE, serious adverse event; SD, stable disease; SLD, sum of the longest diameter; SS, synovial sarcoma; WK, week

Supplementary Table 6. Summary of overall response rate and best overall response (modified intent-to-treat population; N=38)

Parameter/Category	All Tumors	
	Group 3 + Expansion (N=32)	Overall (N=38)
Best Overall Response, n (%)		
Complete Response	0	0
PR	9 (28.1)	9 (23.7)
SD	14 (43.8)	19 (50.0)
PD	6 (18.8)	7 (18.4)
Not Evaluable	3 (9.4)	3 (7.9)
Overall Response Rate		
Complete Response + PR, n (%)	9 (28.1)	9 (23.7)
95% confidence interval	(13.7, 46.7)	(11.4, 40.2)
Disease Control Rate		
Complete Response + PR + SD, n (%)	23 (71.9)	28 (73.7)

Evaluated using RECIST v1.1. LD, lymphodepletion; NSCLC, non−small cell lung cancer; PD, progressive disease; PR, partial response; RECIST, Response Evaluation Criteria in Solid Tumors; SD, stable disease; SS, synovial sarcoma. The 9 patients with PR were all in the expansion group that was composed of 7 patients with SS and 1 patient each with head and neck cancer and NSCLC. Two of the patients with SS received the higher dose of LD chemotherapy. Of the 19 patients with SD, 12 were in the expansion group that was composed of 7 patients with SS (2 of the SS patients received high dose LD chemotherapy), 2 patients with myxoid/round cell liposarcoma, and 1 patient each head and neck, ovarian, and urothelial cancer. Two of the patients with SD were in Group 1 and 3 patients were in Group 2; all had ovarian cancer. Two of the patients with SD were in Group 3: 1 patient with SS and 1 patient with ovarian cancer. Of the 7 patients with PD, 6 were in the expansion group and 1 was an ovarian cancer patient in Group 1. The patients with PD in the expansion group included 2 patients with squamous cell carcinoma (1 squamous cell carcinoma patient received high dose LD chemotherapy) and 1 patient each with SS, gastric cancer, urothelial cancer, and melanoma. LD Chemotherapy Dose and Median Transduced Cell Dose per Group: Group 1: LD chemotherapy dose-cyclophosphamide (600 mg/m^2/day) on Days -7, -6, and -5; fludarabine (30 mg/m^2/day) on Days -7, -6, and -5; median transduced cell dose of 0.1×10^9 cells. Group 2: LD chemotherapy dose-cyclophosphamide (600 mg/m^2/day) on Days -7, -6, and -5; fludarabine (30 mg/m^2/day) on Days -7, -6, and -5; median transduced cell dose of 1.2×10^9 cells. Group 3: LD chemotherapy dose-cyclophosphamide (600 mg/m^2/day) on Days -7, -6, and -5; fludarabine (30 mg/m^2/day) on Days -7, -6, -5, and -4; median transduced cell dose of 5.67×10^9 cells. Expansion: LD chemotherapy dose-cyclophosphamide (600 mg/m^2/day) on Days -7, -6, and -5; fludarabine (30 mg/m^2/day) on Days -7, -6, -5, and -4; median transduced cell dose of 7.85×10^9 cells. Expansion: Higher dose LD chemotherapy-cyclophosphamide (1800 mg/m^2/day) for Days -3 and -2 in combination with fludarabine (30 mg/m^2/day) Days -5, -4, -3, and -2; median transduced cell dose of 7.85×10^9 cells

Supplementary Table 7. Integration site analysis to evaluate clonality status

Patient	Sample	Unique Sites	Pielou Diversity Index	Persistence (Vector Copies/μg DNA)
1	T-cells	19,546	1.000	
	Month 6 PBMC	2,190	1.000	27,962
	Month 12 PBMC	252	0.989	4,675
2	T-cells	6,558	1.000	
	Month 9 PBMC	976	1.000	30,501
	Month 12 PBMC	850	0.995	24,550
3	T-cells	1,559	1.000	
	Month 9 PBMC	1,307	1.000	34,445
	Month 12 PBMC	551	0.997	57,181
4	T-cells	2,369	1.000	
	Month 12 PBMC	125	0.998	12,895
	Month 15 PBMC	1,015	0.993	11,011
5	T-cells	5,574	1.000	
	Month 6 PBMC	1,182	0.999	81,859
	Month 12 PBMC	381	0.999	12,876

Integration site analysis was used to evaluate clonality status. The Pielou evenness index (between 0 and 1, 1 meaning that the distribution of the cells is completely even across all integration sites, i.e., no clonal dominance, and the vector copy number in the corresponding PBMC sample demonstrate a high level of polyclonality and absence of clonal dominance in all post-infusion samples in patients with elevated long-term persistence. PBMC, peripheral blood mononuclear cell

Supplementary Table 8. Serum proteins peak and AUC concentrations for patients with cytokine release syndrome versus patients without cytokine release syndrome

**Serum protein**	**Metric**[a]	**Median pg/mL**		**Univariate *P* value**[b]		**Multiple comparisons *P* adjusted**[c]	
		**CRS**	**Non-CRS**				
GMCSF	Peak	7.88	1.725	0.000971	***	0.015529	*
	AUC	39.88	12.156	0.000221	***	0.004191	**
IFNγ	Peak	270.83	47.68	0.00012	***	0.002402	**
	AUC	1455.046	467.0139	0.00061	***	0.010976	**
IL-10	Peak	9.785	2.915	0.020994	*	0.230935	ns
	AUC	96.38442	28.0825	0.023466	*	0.234658	ns
IL-2	Peak	2.83	2.04	0.052278	ns	0.431994	ns
	AUC	28.96357	15.46125	0.001245	**	0.018675	*
IL-2Rα	Peak	7758.99	5300.86	0.057269	ns	0.431994	ns
	AUC	99860.57	62037.02	0.058604	ns	0.431994	ns
IL-5	Peak	39.88	6.59	0.018256	*	0.21907	ns
	AUC	229.0675	36.0775	0.004776	**	0.062088	ns
IL-6	Peak	156.56	19.59	0.001374	**	0.01924	*
	AUC	1168.038	132.966	0.000777	***	0.013202	*
IL-8	Peak	69.08	119.33	0.518362	ns	1	ns
	AUC	870.515	689.9	0.631126	ns	1	ns
IL-15	Peak	49.26	43.57	0.304173	ns	0.91252	ns
	AUC	569.15	375.3841	0.071126	ns	0.431994	ns
TNFα	Peak	7.76	5.415	0.047999	*	0.431994	ns
	AUC	98.18857	78.77875	0.088132	ns	0.431994	ns

[a] 'Peak' represents the maximum pg/mL measured across post-infusion sampling time points collected across first 21 days post-infusion; 'AUC' calculated 0 to 21 days post-infusion
[b] Two-sided Wilcoxon rank sum test
[c] Adjusted *P* value by Holm method
AUC, area under the curve; CRS, cytokine release syndrome; GMCSF, granulocyte-macrophage colony-stimulating factor; IFN, interferon; IL, interleukin; ns, not significant; TNF, tumor necrosis factor
**P*<0.05; ***P*<0.01; ****P*<0.001; *****P*<0.0001

Supplementary Table 9. Correlation of peak serum level with best percentage change in target lesion SLD

Serum protein	Two-sided Spearman R	*P* value	
IFN-gamma	-0.626	0.00008	****
ARG1	0.556	0.00065	***
CX3CL1	-0.482	0.00377	**
IL6	-0.458	0.00616	**
CXCL10	-0.456	0.00637	**
CXCL9	-0.434	0.00971	**
CCL3	-0.424	0.01177	*
CCL23	-0.421	0.01238	*
IL10	-0.394	0.02000	*
MCP-4	-0.389	0.02165	*
MCP-3	-0.375	0.02721	*
TNF	-0.365	0.03176	*
MCP-1	-0.364	0.03204	*
HO-1	-0.345	0.04311	*

OLINK Normalized Protein eXpression (NPX) unit measured across all available post-infusion sampling time points with best % change in target lesion SLD
*P<0.05; **P<0.01; ***P<0.001; ****P<0.0001
SLD, sum of longest diameters

Supplementary Table 10. Afami-cel dose, vector copy number, tumor MAGE-A4 expression and serum IFNγ profiles in ovarian cancer across all dose groups

	Transduced Dose	Peak VCN/µg DNA	MAGE-A4 Expression		IFNγ	
			H Score	% 2+/3+	Peak (units)	AUC (units)
Dose Group 1	1.00E+08	6307.9	15	5	33.8	465.3
	1.00E+08	24889.1	130	50	139.7	1018.6
	1.00E+08	1101.7	18	6	25	292.3
Dose Group 2	1.20E+09	26731.9	145	60	79.8	724.5
	1.20E+09	121899.7	75	25	67.4	468.7
	1.19E+09	118069.2	165	55	293.1	2465.8
Group 3/Expansion	5.68E-09	129581.3	18	6	108.6	502.7
	9.43E+09	315990.1	220	70	121.3	662.6
	7.85E+09	312971.3	55	10	82.7	NE

AUC, area under the curve (Day 0-Day 21); IFN, interferon; MAGE-A4, melanoma-associated antigen A4; NE, non-evaluable as sampling timepoints only available to Day 8; VCN, vector copy number

Supplementary Table 11. Laboratory values to define adequate organ function

System	Laboratory value
**Hematological**	
Absolute neutrophil count	≥1.5 × 10^9/L (without granulocyte colony-stimulating factor support)
Platelets	≥100 × 10^9/L
Hemoglobin	>80 g/L (without transfusion support within 7 days prior to leukapheresis)
**Coagulation**	
Prothrombin time or international normalized ratio	≤1.5 × ULN unless receiving therapeutic anticoagulation
Partial thromboplastin time	≤1.5 × ULN unless receiving therapeutic anticoagulation
**Renal**	
Calculated or measured creatinine clearance[a]	≥60 ml/min<br>Exception: patients with urothelial cancer ≥40 ml/min
**Hepatic**	
Serum total bilirubin	≤1.5 × ULN (unless patient had documented Gilbert's syndrome)
Alanine aminotransferase/serum glutamic pyruvic transaminase	≤2.5 × ULN

Renal function was reassessed at baseline

[a]Creatinine clearance was calculated using the Cockcroft-Gault Method:

$$\text{Creatinine clearance} = \frac{(140 - \text{age}) \times \text{weight kg}}{72 \times \text{serum creatinine mg/dl}} (\times 0.85 \text{ in females})$$

or by 24-hour urine creatinine collection or by nuclear medicine ethylenediamine tetraacetic acid glomerular filtration rate measurement, according to standard practice at the treating institution

ULN, upper limit of normal

Supplementary Table 12. Prohibited therapy/treatment prior to leukapheresis or lymphodepleting chemotherapy

**Treatment/therapy**	**Required washout prior to leukapheresis**	**Required washout prior to lymphodepletion**
Cytotoxic chemotherapy	3 weeks	3 weeks
Small molecules/tyrosine kinase inhibitor such as dabrafenib, trametinib, vemurafaneb, and cobimetinib. Note: no washout period was required for compounds that do not cause bone marrow suppression/lymphopenia or for EGFR and ALK/ROS-1 inhibitors unless the multi-kinase inhibitor targets VEGFR (for example, afatinib), PDGFR, or c-Kit receptors	1 week	1 week
Immune therapy (including monoclonal antibody therapy, checkpoint inhibitors, and biologics)	2 weeks	2 weeks
Experimental anticancer vaccine	N/A	2 months in the absence of tumor response<br>The patient should have been excluded if their disease was responding to an experimental vaccine given within 6 months
Gene therapy using an integrating vector	Any use of previous gene therapy using an integrating vector was not permitted	Any use of previous gene therapy using an integrating vector was not permitted
Corticosteroids or any other immunosuppressive therapy. Note: use of topical steroids and inhaled steroids was not an exclusion	2 weeks	2 weeks
Investigational treatment	2 weeks or five half-lives, whichever was shorter	2 weeks or five half-lives, whichever was shorter
Radiotherapy that involved the lung (V20 exceeding 30% lung volume) or pericardium (>20 Gy). Note: exception for a lesser dose or radiation exposure to lung/mediastinum than stated, administered within 4 weeks prior to	N/A	3 months

Treatment/therapy	Required washout prior to leukapheresis	Required washout prior to lymphodepletion
lymphodepletion. Electron beam radiotherapy to superficial structures in the chest was permitted		
Radiation to vital organs (for example, liver and kidney)	N/A	4 weeks
Radiation to the pelvis	4 weeks	4 weeks
Whole brain radiotherapy or brain stereotactic radiosurgery	N/A	2 weeks
Radiotherapy to the target lesions	N/A	3 months prior to lymphodepleting chemotherapy<br>A lesion with unequivocal progression may have been considered a target lesion.<br>Note: there was no washout period for palliative radiation to non-target organs
Note: duration of any other anticancer therapies must have been discussed with the sponsor study physician<br>ALK/ROS1, anaplastic lymphoma kinase/proto-oncogene tyrosine-protein kinase; EGFR, estimated glomerular filtration rate; N/A, not applicable; PDGFR, platelet-derived growth factor receptor; VEGFR, vascular endothelial growth actor receptor		

Supplementary Table 13. List of institutional review boards and ethics committees

Site	Investigator	Institutional review board or ethics committee	Chairperson
Duke University Medical Center	Jeffrey Clarke, MD	Duke University Health System Institutional Review Board<br>2424 Erwin Road<br>Duke University Medical Center, Suite 405, Box 2712<br>Durham, NC 27705	Jody Power
The University of Texas MD Anderson Cancer Center	David S. Hong, MD	The University of Texas MD Anderson Cancer Center<br>Institutional Review Board 7007 Bertner Avenue, Unit 1637<br>Houston, TX 77030	Dr. Jennifer Litton, M.D.
H. Lee Moffitt Cancer Center and Research Institute	Mihaela Druta, MD	Advarra IRB<br>6940 Columbia Gateway Drive Suite 110<br>Columbia MD 21046	Advarra: Tony Davis
Sylvester Comprehensive Cancer Center	Brian Matthew Slomovitz, MD	University of Miami Institutional Review Board<br>1400 NW 10th Avenue, Suite 1200A<br>Miami, FL 33136	Daniel H. Kett
Washington University School of Medicine	Brian Van Tine, MD	Western Institutional Review Board (WIRB)<br>1019 39th Avenue SE Suite 120<br>Puyallup, WA 98374	Donald D. Deieso
Princess Margaret Cancer Center	Dr. Marcus Butler	University Health Network<br>Research Ethics Board<br>700 University Avenue Hydro Building, 10th Floor Suite 1056 Toronto, Ontario M5D 1Z5	Dr. David Hogg

Site	Investigator	Institutional review board or ethics committee	Chairperson
James Cancer Hospital and Solove Research Institute	David A. Liebner, MD	The Ohio State University Cancer Institutional Review Board Office of Responsible Research Practices<br>1960 Kenny Road<br>300 OSU Research Foundation<br>Columbus, OH 43210	William Carson III, MD
The Sarah Cannon Research Institute	Melissa L. Johnson, MD	Western Institutional Review Board<br>1019 39th Avenue SE Suite 120<br>Puyallup, WA 98374	Donald D. Deieso
Fox Chase Cancer Center	Anthony Olszanski, MD	WIRB<br>1019 39th Avenue SE Suite 120<br>Puyallup, WA 98374	Donald D. Deieso
Roswell Park Cancer Institute	Adekunle Odunsi, MD, PhD	Roswell Park Institute<br>Institutional Review Board<br>Elm & Carlton Streets<br>Buffalo, New York 14263	Donald Handley

Supplementary Table 14. Fludarabine and cyclophosphamide treatment schema: all dose groups

Dose Group	Afami-cel Dose Range	Lymphodepletion Regimen
Dose Group 1	$0.08\text{-}0.12 \times 10^9$	Cyclophosphamide (600 mg/m^2/day) plus fludarabine (30 mg/m^2/day) on Days -7, -6, and -5
Dose Group 2	$0.5\text{-}1.2 \times 10^9$	Cyclophosphamide (600 mg/m^2/day) plus fludarabine (30 mg/m^2/day) on Days -7, -6, and -5
Dose Group 3	$1.2\text{-}6 \times 10^9$	Cyclophosphamide (600 mg/m^2/day) on Days -7, -6, and -5 plus fludarabine (30 mg/m^2/day) on Days -7, -6, -5, and -4
Dose Expansion Group	$1.2\text{-}10 \times 10^9$	Cyclophosphamide (1800 mg/m^2/day) on Days -3 and -2 plus fludarabine (30 mg/m^2/day) on Days -5, -4, -3, and -2
		Cyclophosphamide (600 mg/m^2/day) on Days -7, -6, and -5 plus fludarabine (30 mg/m^2/day) Days -7, -6, -5, and -4

Supplementary Table 15. Antibody dilutions

**Reagent**	**Clone**	**Supplier**	**Titer (µl)**	**Dilution**
Live/Dead Aqua		Fisher Scientific	0.25	200
CD3 BUV395	SK7	BD Biosciences	0.50	100
CD4 BUV496	SK3	BD Biosciences	0.50	100
CD8 BUV737	SK1	BD Biosciences	1.00	50
CD45RA APC-Cy7	HI100	Biolegend	0.25	200
CD197 (CCR7) PE-Cy7	G043H7	Biolegend	1.00	50
Dextramer PE : MAGE A-4		Immudex	10.0	5
The titer column shows volume used in 50 µl total staining volume, and the dilution column shows calculated dilution factor				
