# Supplementary material for: Autologous T cell therapy for MAGE-A4+ solid cancers in HLA-A*02+ patients: a phase 1 trial
Source: Nat Med. 2023 Jan 9;29(1):104–14. doi: 10.1038/s41591-022-02128-z (PMC9873554; doi:10.1038/s41591-022-02128-z)
Supplement: Supplementary file 2 — Reporting Summary [file 41591_2022_2128_MOESM2_ESM.pdf]

nature portfolio

Corresponding author(s): David S. Hong

Last updated by author(s): Nov 7, 2022

Reporting Summary

Nature Portfolio wishes to improve the reproducibility of the work that we publish. This form provides structure for consistency and transparency in reporting. For further information on Nature Portfolio policies, see our Editorial Policies and the Editorial Policy Checklist.

Statistics

For all statistical analyses, confirm that the following items are present in the figure legend, table legend, main text, or Methods section.

n/a	Confirmed	
☐	☒	The exact sample size (n) for each experimental group/condition, given as a discrete number and unit of measurement
☒	☐	A statement on whether measurements were taken from distinct samples or whether the same sample was measured repeatedly
☐	☒	The statistical test(s) used AND whether they are one- or two-sided<br>*Only common tests should be described solely by name; describe more complex techniques in the Methods section.*
☒	☐	A description of all covariates tested
☒	☐	A description of any assumptions or corrections, such as tests of normality and adjustment for multiple comparisons
☐	☒	A full description of the statistical parameters including central tendency (e.g. means) or other basic estimates (e.g. regression coefficient) AND variation (e.g. standard deviation) or associated estimates of uncertainty (e.g. confidence intervals)
☐	☒	For null hypothesis testing, the test statistic (e.g. F, t, r) with confidence intervals, effect sizes, degrees of freedom and P value noted<br>*Give P values as exact values whenever suitable.*
☒	☐	For Bayesian analysis, information on the choice of priors and Markov chain Monte Carlo settings
☒	☐	For hierarchical and complex designs, identification of the appropriate level for tests and full reporting of outcomes
☐	☒	Estimates of effect sizes (e.g. Cohen's d, Pearson's r), indicating how they were calculated

Our web collection on statistics for biologists contains articles on many of the points above.

Software and code

Policy information about availability of computer code

Data collection	BD FACSDiva 9.0; Sartorius Incucyte Zoom software 2019B Rev2; MSD SQ120 reader; Fluidigm® Biomark™ system; Indica Labs HALO® image analysis software.
Data analysis	SAS version 9.4; BD FlowJo 9.9; Sartorius Incucyte Zoom software 2019B Rev2; Indica Labs HALO® image analysis software and ISH version 3.3.9 algorithm; R-studio (and R-4.2.1 version), ComplexHeatmap and appropriate R script packages.

For manuscripts utilizing custom algorithms or software that are central to the research but not yet described in published literature, software must be made available to editors and reviewers. We strongly encourage code deposition in a community repository (e.g. GitHub). See the Nature Portfolio guidelines for submitting code & software for further information.

Data

Policy information about availability of data

All manuscripts must include a data availability statement. This statement should provide the following information, where applicable:

- Accession codes, unique identifiers, or web links for publicly available datasets
- A description of any restrictions on data availability
- For clinical datasets or third party data, please ensure that the statement adheres to our policy

The NanoString data are available publicly at: https://www.ncbi.nlm.nih.gov/geo/query/acc.cgi?acc=GSE202156. The clinical datasets generated during and/or analyzed during the current study are available upon request from the corresponding author for research only, non-commercial purposes. Such datasets include

study protocol, SAP, individual participant data that underlie the results reported in this article after de-identification (text, tables, figures and appendices), as well as supporting documentation as required. Restrictions relating to patient confidentiality and consent will be maintained by aggregating and anonymizing identifiable patient data. The clinical data will be available beginning immediately following article publication and thereafter with no time limit. Requests should be sent in writing describing the nature of the proposed research and extent of data requirements. Data recipients are required to enter a formal data sharing agreement that describes the conditions for release and requirements for data transfer, storage, archiving, publication and intellectual property. Requests should be directed to Dennis Williams, PharmD, and will be reviewed by the corresponding senior authors DSH and MOB. and by Adaptimmune. Responses will typically be provided within 60 days of the initial request.

Human research participants

Policy information about studies involving human research participants and Sex and Gender in Research.

Reporting on sex and gender

This first-in-human study included a 3+3 dose-escalation scheme designed to understand the optimal dose among patients, with biological characteristics such as age, weight, and sex inherently considered due to the nature of the study. Representatives of both sexes (male n=22, 57.9%; female, n=16, 42.1%) were included in the trial (reported in Table 1). Sex was determined based on self-reporting; patients were asked to check a box indicating their biological sex with only two options (ie., male or female). Gender information was not collected. No sex-based analyses were performed as there were no apparent differences in optimal dose or safety between males and females.

Population characteristics

Population demographics are reported in Table 1 and Supplementary Table 2. A brief summary is as follows: adult patients, aged 18-75 with advanced solid cancers across 9 tumor types, previously treated with SOC agents, Eastern Cooperative Oncology Group (ECOG) score of O or 1, measurable disease per RECIST vl.l, adequate organ function including creatinine clearance ≥60 ml/min, and HLA-A*02 positive with tumor. expression of MAGE-A4 antigen. As mentioned below, covariates were not controlled because the primary objective was safety and not efficacy. Observed treatment effects are being further explored in the Phase 2 SPEARHEAD-1 trial. In addition, the study was not powered for either safety or efficacy; hence the data are summarized descriptively.

Recruitment

Patients were recruited from 05July2017 to 11Nov2019 by participating investigators from a screening study initiated to pre-screen patients with advanced solid tumors for the presence of inclusion and exclusion alleles (NCT02636855). A total of 854 HLA-A*02-eligible patients proceeded to tumor MAGE-A4 testing, of which 225 were MAGE-A4 positive. Eligibility criteria included age ≥18 to ≤75 years, histologically confirmed cancer diagnosis, and measurable disease according to Response Evaluation Criteria in Solid Tumors version 1.1 before lymphodepleting (LD) chemotherapy. No self-selection or other biases are expected as the inclusion and exclusion criteria detail eligibility for enrollment.

Ethics oversight

Patients had voluntarily agreed to participate by giving written informed consent in accordance with International Council on Harmonization (ICH) Good Clinical Practice (GCP) guidelines and applicable local regulations. No compensation was provided for study participation. Participants may have received reimbursement for any costs incurred as a results of study participation.

The final study protocol and subject informed consent documentation was approved by the Institutional Review Board (IRB)/ Independent Ethics Committee (IEC) and any other site level committee deemed appropriate by the 10 institutions listed below. Approval from each applicable committee was received in writing before initiation of the study. Below lists the institutional review boards and ethics committees.

Site: Duke University Medical Center
Investigator: Jeffrey Clarke, MD
Institutional review board or ethics committee: Duke University Health System Institutional Review Board, 2424 Erwin Road, Duke University Medical Center, Suite 405, Box 2712, Durham, NC 27705
Chairperson: Jody Power

Site: The University of Texas MD Anderson Cancer Center
Investigator: David S. Hong, MD
Institutional review board or ethics committee: The University of Texas MD Anderson Cancer Center, Institutional Review Board 7007 Bertner Avenue, Unit 1637, Houston, TX 77030
Chairperson: Dr. Jennifer Litton, M.D.

Site: H. Lee Moffitt Cancer Center and Research Institute
Investigator: Mihaela Druta, MD
Institutional review board or ethics committee: Advarra IRB, 6940 Columbia Gateway Drive Suite 110, Columbia MD 21046
Chairperson: Advarra, Tony Davis

Site: Sylvester Comprehensive Cancer Center
Investigator: Brian Matthew Slomovitz, MD
Institutional review board or ethics committee: University of Miami Institutional Review Board, 1400 NW 10th Avenue, Suite 1200A, Miami, FL 33136
Chairperson: Daniel H. Kett

Site: Washington University School of Medicine
Investigator: Brian Van Tine, MD
Institutional review board or ethics committee: Western Institutional Review Board (WIRB), 1019 39th Avenue SE Suite 120, Puyallup, WA 98374
Chairperson: Donald D. Deieso

Site: Princess Margaret Cancer Center
Investigator: Dr. Marcus Butler
Institutional review board or ethics committee: University Health Network, Research Ethics Board , 700 University Avenue Hydro Building, 10th Floor Suite 1056 Toronto, Ontario M5D 1Z5
Chairperson: Dr. David Hogg

Site: James Cancer Hospital and Solove Research Institute
Investigator: David A. Liebner, MD
Institutional review board or ethics committee: The Ohio State University Cancer Institutional Review Board Office of Responsible Research Practices, 1960 Kenny Road, 300 OSU Research Foundation, Columbus, OH 43210
Chairperson: William Carson III, MD

Site: The Sarah Cannon Research Institute
Investigator: Melissa L. Johnson, MD
Institutional review board or ethics committee: Western Institutional Review Board, 1019 39th Avenue SE Suite 120, Puyallup, WA 98374
Chairperson: Donald D. Deieso

Site: Fox Chase Cancer Center
Investigator: Anthony Olszanski, MD
Institutional review board or ethics committee: WIRB, 1019 39th Avenue SE Suite 120, Puyallup, WA 98374
Chairperson: Donald D. Deieso

Site: Roswell Park Cancer Institute
Investigator: Adekunle Odunsi, MD, PhD
Institutional review board or ethics committee: Roswell Park Institute Institutional Review Board, Elm & Carlton Streets, Buffalo, New York 14263
Chairperson: Donald Handley

Note that full information on the approval of the study protocol must also be provided in the manuscript.

Field-specific reporting

Please select the one below that is the best fit for your research. If you are not sure, read the appropriate sections before making your selection.

☒ Life sciences ☐ Behavioural & social sciences ☐ Ecological, evolutionary & environmental sciences

For a reference copy of the document with all sections, see nature.com/documents/nr-reporting-summary-flat.pdf

Life sciences study design

All studies must disclose on these points even when the disclosure is negative.

Sample size	This is a first-in-human, open-label, single arm, Phase 1, safety and dose finding study. The sample size was based on clinical judgment. The study was not powered for either safety or efficacy and hence the data are summarized descriptively. The study used a modified 3 + 3 cell dose escalation design to evaluate dose-limiting toxicities and determine the target cell dose range. Following the dose escalation phase, up to 30 patients total at the selected dose range (inclusive of patients accrued during the dose escalation) were treated across all the eligible tumor types in the dose expansion phase, to characterize and better assess overall safety and anti-tumor activity. Up to an additional 10 were treated at the selected dose range in a radiation sub-study.
Data exclusions	No data were excluded.
Replication	Replication is not applicable because this is a Phase 1 safety and dose-finding study. However, further assessment of outcomes following intervention with afami-cel is near completion in the Phase 2 SPEARHEAD-1 trial.
Randomization	Randomization is not applicable because this is an open-label, single arm, Phase 1 safety and dose finding study. Covariates were not controlled because the primary objective was safety and not efficacy. In addition, the study was not powered for either safety or efficacy; hence the data are summarized descriptively. Observed treatment effects are being further explored in the Phase 2 SPEARHEAD-1 trial.
Blinding	Blinding is not applicable because this is an open-label, single arm study.

Reporting for specific materials, systems and methods

We require information from authors about some types of materials, experimental systems and methods used in many studies. Here, indicate whether each material, system or method listed is relevant to your study. If you are not sure if a list item applies to your research, read the appropriate section before selecting a response.

Materials & experimental systems

n/a	Involved in the study
☐	☒ Antibodies
☒	☐ Eukaryotic cell lines
☒	☐ Palaeontology and archaeology
☒	☐ Animals and other organisms
☐	☒ Clinical data
☒	☐ Dual use research of concern

Methods

n/a	Involved in the study
☒	☐ ChIP-seq
☐	☒ Flow cytometry
☒	☐ MRI-based neuroimaging

Antibodies

Antibodies used

Reagent; Clone; Supplier Titer (µl); Dilution:

1) Live/Dead Aqua; Fisher Scientific; 0.25; 200

2) CD3 BUV395; SK7; BD Biosciences; 0.50; 100

3) CD4 BUV496; SK3; BD Biosciences; 0.50; 100

4) CD8 BUV737; SK1 BD Biosciences; 1.00; 50

5) CD45RA APC-Cy7; HI100 Biolegend; 0.25; 200

6) CD197 (CCR7) PE-Cy7; G043H7; Biolegend; 1.00; 50

7) Dextramer PE:MAGE A-4; Immudex; 10.0; 5

Validation

All antibodies are validated for specificity to their respective target on human cells as detailed by the manufacturers.

Clinical data

Policy information about clinical studies

All manuscripts should comply with the ICMJE guidelines for publication of clinical research and a completed CONSORT checklist must be included with all submissions.

Clinical trial registration

NCT03132922

Study protocol

Provided with the submission but not to be published.

Data collection

Data were collected at medical centers and hospitals at 10 sites across North America (USA, 9 sites, 28 patients; Canada, 1 site, 10 patients). Recruitment took place between 05July2017 and 11Nov2019, and data were collected until 01Sept2020.

Investigator; Location; Number of Patients Treated

David S Hong, MD;The University of Texas MD Anderson Cancer Center, Houston, TX; 16

Marcus Butler, MD; Princess Margaret Cancer Centre, Toronto, ON, Canada; 10

Brian Van Tine, MD; Washington University School of Medicine, St. Louis, MO; 7

Anthony Olszanski, MD; Fox Chase Cancer Center, Philadelphia, PA; 2

David A Liebner, MD; James Cancer Hospital and Solove Research Institute, Columbus, OH; 1

Melissa L Johnson, MD; The Sarah Cannon Research Institute, Nashville, TN; 1

Adekunle Odunsi, MD, PhD; Roswell Park Cancer Institute, Buffalo, NY; 1

Jeffrey Clarke, MD; Duke University Medical Cancer Center, Durham, NC; 0

Mihaela Druta, MD; H. Lee Moffitt Cancer and Research Institute, Tampa, FL; 0

Brian M Slomovitz, MD; Sylvester Comprehensive Cancer Center, Miami, FL; 0

Outcomes

The primary objective was evaluation of safety and tolerability with endpoints including TEAEs, serious adverse events (SAE), DLTs, and detection of replication competent lentivirus (RCL). Secondary endpoints included overall response rate (ORR) confirmed by RECIST vl.l, best overall response (BoR), time to response (TTR), duration of response (DoR), duration of stable disease (DosD), progression-free survival (PFS), and overall survival (OS). Exploratory objectives included evaluation of cell persistence and cytokines.

Flow Cytometry

Plots

Confirm that:

☒ The axis labels state the marker and fluorochrome used (e.g. CD4-FITC).

☒ The axis scales are clearly visible. Include numbers along axes only for bottom left plot of group (a 'group' is an analysis of identical markers).

☒ All plots are contour plots with outliers or pseudocolor plots.

☒ A numerical value for number of cells or percentage (with statistics) is provided.

Methodology

Sample preparation	Cryopreserved manufactured product (MP) and peripheral blood mononuclear cells (PBMC) from human clinical trial subjects were thawed, stained and washed for immunophenotypic profiling using multicolor staining panels (CD3 (SK7), CD4 (SK3), CD8 (SK1), BD Biosciences, Franklin Lakes, NJ, USA; CD45RA (HI100), CCR7 (G043H7), Biolegend, San Diego, CA, USA; Live/Dead Fix Aqua, Fisher Scientific, Waltham, MA, USA). For all samples, in the CD3+ live population, subsets of CD4+ and CD8+ cells (assessed for transduction using an MHC dextramer reagent were further classified into memory subtypes by expression of CCR7 and CD45RA.
Instrument	BD LSR Fortessa
Software	FlowJo
Cell population abundance	No sorting was performed
Gating strategy	FSC/SSC gating on lymphocytes, viability dye dead cell exclusion gate and gates for all single markers were set in relation to FMO (fluorescence minus one) controls

☒ Tick this box to confirm that a figure exemplifying the gating strategy is provided in the Supplementary Information.
